# DHA: Nutritional Programming During the First 1000 Days of Life

**DOI:** 10.3390/nu18081178

**Published:** 2026-04-09

**Authors:** Laura Maria Sollena, Maurizio Carta, Vincenzo Insinga, Bruna Gabriele, Veronica Notarbartolo, Costanza Sortino, Mario Giuffrè

**Affiliations:** 1 Neonatal Intensive Care Unit, Department of Health Promotion, Mother and Child Care, Internal Medicine and Medical Specialties “G. D’Alessandro”, University of Palermo, 90127 Palermo, Italy; lauramaria.sollena@community.unipa.it (L.M.S.); costanza.sortino@community.unipa.it (C.S.); mario.giuffre@unipa.it (M.G.); 2 Neonatology and Neonatal Intensive Care Unit, University Hospital Policlinico “Paolo Giaccone”, 90127 Palermo, Italy; vincenzo.insinga@policlinico.pa.it (V.I.); bruna.gabriele@policlinico.pa.it (B.G.)

**Keywords:** first 1000 days of life, docosahexaenoic acid, DHA, LCPUFA, *n*-3 fatty acid, pregnancy, lactation, newborn, preterm infant, neurodevelopment, immune system

## Abstract

Background: The first 1000 days of life, from conception to 2 years of age, represent a critical window during which nutrition can exert long-lasting effects on neurodevelopment, immune maturation, and susceptibility to prematurity-related morbidity. Docosahexaenoic acid (DHA) is a key structural *n*-3 long-chain polyunsaturated fatty acid of the brain and retina, characterized by rapid fetal accretion during the third trimester. Methods: We conducted a narrative review of studies published from March 2015 up to December 2025, including randomized controlled trials, follow-up studies, and systematic reviews/meta-analyses about DHA supplementation during pregnancy, lactation, infancy and early childhood, and its role on development. Results: Across the first 1000 days, DHA supplementation improves biochemical DHA status, particularly in populations with low baseline levels (moderate to high level of evidence), while clinical outcomes remain heterogeneous. During pregnancy, some benefits in specific cognitive and behavioral domains have been demonstrated, whereas effects on global cognition and long-term behavior are frequently null (moderate evidence). Visual outcomes appear favorable, with improvements in visual acuity (moderate evidence). In preterm infants, enteral DHA—often combined with arachidonic acid (ARA)—is feasible and well tolerated. DHA may reduce inflammatory markers and necrotizing enterocolitis risk when in equilibrium with ARA (low to moderate evidence), while no evidence supports the link between DHA and reduced risk of bronchopulmonary dysplasia and retinopathy of prematurity (moderate evidence). Neurodevelopmental outcomes are mixed: neuroimaging studies suggest enhanced white matter maturation with DHA + ARA, whereas most trials show no clear benefit regarding standardized developmental scores (moderate evidence). Conclusions: DHA is biologically essential during the first 1000 days, but its clinical impact depends on timing, dose, baseline status, and prematurity-related context. The balance between DHA and ARA, rather than DHA supplementation alone, emerges as a key determinant of clinical efficacy, supporting a shift toward precision-based nutritional strategies in early life.

## 1. Introduction

The first 1000 days of life—from conception and pregnancy through the first 2 years—represent a period often described as a “window of opportunity” to promote better developmental outcomes, but it can also be considered a “window of susceptibility,” during which adverse epigenetic influences, an increased risk of disease development and nutritional deficiency may occur [1].

The essential role of nutrition on brain development during the first 1000 days is widely recognized [2]. During this window, fetuses, infants and children undergo particular physiological changes and consequently have well-defined nutritional needs.

Docosahexaenoic acid (DHA) is a key structural component of neuronal cell membranes and of the rod outer segments of the retina, and it is widely recognized as essential for normal neural, auditory, olfactory and visual function. DHA plays an important role in brain maturation, cognitive development, and overall health [3,4].

DHA is the main *n*-3 fatty acid in the fetal brain, crucial for neurogenesis, synaptogenesis, neuroinflammation, and neurovascular coupling. The third trimester represents a critical period marked by a substantial increase [5]. Importantly, DHA metabolism is closely linked to *n*-6 fatty acids metabolism, particularly that of ARA, as both compete for shared enzymatic pathways. This interaction influences their relative incorporation into cell membranes and downstream effects on inflammation and neurodevelopment. Therefore, the balance between DHA and ARA represents a critical factor in modulating these biological processes and their clinical implications.

This narrative review explores potential early-life DHA supplementation. Specifically, it aims to evaluate the physiological role of DHA during the first 1000 days of life through its structural role in cell membranes and its involvement in the key developmental processes mentioned above, the clinical impact of DHA supplementation in this critical window and how alterations in DHA status may contribute to clinical outcomes (e.g., neurodevelopment, immune system development, and risk of BPD, NEC, and ROP in preterm infants).

## 2. Materials and Methods

A literature search was conducted to identify relevant studies evaluating the role of docosahexaenoic acid (DHA) and its supplementation during the first 1000 days of life, from conception to 2 years of age. The search covered publications from March 2015 up to December 2025 and was performed in the following databases: PubMed, Cochrane Library, Scopus, and Google Scholar. Google Scholar was used as a complementary search tool to identify additional studies, including published articles not indexed in the primary databases.

Search terms were combined using Boolean operators, MeSH terms and free-text keywords related to DHA and early-life nutrition. Key words included: “docosahexaenoic acid”, “DHA”, “long-chain polyunsaturated fatty acids”, “*n*-3 fatty acids”, “first 1000 days”, “pregnancy”, “lactation”, “preterm infants”, “neurodevelopment”, “visual development”, “immune system”.

Studies were considered eligible if they evaluated DHA supplementation or DHA status during the first 1000 days of life, including pregnancy, lactation, infancy, and early childhood up to 2 years of age. We included studies investigating DHA administered either alone or in combination with other nutrients (particularly arachidonic acid (ARA) and micronutrients) provided that DHA exposure represented a central component of the intervention or analysis.

Eligible studies included randomized controlled trials, follow-up studies, systematic reviews and meta-analyses, and observational studies reporting clinical, developmental, or biochemical outcomes associated with DHA exposure. Outcomes of interest included neurodevelopmental outcomes, immune and inflammatory responses, and major preterm morbidities such as necrotizing enterocolitis (NEC), bronchopulmonary dysplasia (BPD), and retinopathy of prematurity (ROP).

Level of evidence (LOE) was qualitatively assigned according to GRADE, used as a conceptual framework rather than a formal systematic grading approach. We consider study design, risk of bias, consistency, and methodological limitations. Randomized controlled trials (RCTs) and meta-analyses were considered moderate- to high-quality evidence depending on the quality of included studies, while observational studies, narrative reviews, and position statements were classified as low-quality evidence. LOE was downgraded when relevant limitations were identified, including lack of blinding, small sample size, or heterogeneity.

Studies were excluded if they did not report outcomes related to DHA exposure during early life, or focused exclusively on adult populations or unrelated nutritional interventions. Articles not available in English were also excluded.

## 3. DHA: Physiology

Long-chain polyunsaturated fatty acids (LCPUFAs) are essential constituents of membrane phospholipids. They are classified into three categories according to the position of the first double bond in the carbon chain: *n*-3, *n*-6, and *n*-9. The major *n*-3 LCPUFAs include α-linolenic acid (ALA), eicosapentaenoic acid (C20:5*n*-3, EPA) and docosahexaenoic acid (C22:6*n*-3, DHA), whereas key members of the *n*-6 family include linoleic acid (LA) and arachidonic acid (ARA) [6].

Humans lack the enzymatic capacity to synthesize α-linolenic acid (ALA) and linoleic acid (LA) de novo; therefore, these fatty acids are considered dietarily essential and must be obtained through the diet. ALA, the precursor for the *n*-3 PUFA family, is found in walnuts and the seeds of flax and chia, while seafood, like fat fish or fish oil, is the major source of EPA and DHA in most industrialized countries [7,8]. Docosahexaenoic acid (DHA) and arachidonic acid (ARA) can also be endogenously synthesized from their respective precursors, ALA and LA, in a pathway involving elongation and desaturation reactions, primarily catalyzed by Δ-5 and Δ-6 desaturase enzymes. However, the efficiency of these metabolic pathways is limited and exhibits interindividual variability influenced by genetic variability, population-specific differences, and epigenetic regulation.

Importantly, genetic polymorphisms in the fatty acid desaturase (FADS) gene cluster can significantly influence desaturase activity, modulating endogenous DHA and ARA synthesis and contributing to variability in LCPUFAs status [9]. Variants such as rs66698963 or rs174570 have been associated with altered FADS1 and FADS2 expression through epigenetic mechanisms, including DNA methylation, linking genetic variability to functional metabolic outcomes [10,11].

These differences reflect diet-driven evolutionary adaptations. Dietary composition further modulates these genetic effects: LA and ALA compete for the same enzymatic pathways, and high dietary LA intake—typical of Western dietary patterns—can reduce the efficiency of conversion of ALA into DHA, particularly in individuals with reduced desaturase activity. Specifically, alleles associated with enhanced LCPUFAs biosynthesis and more efficient FADS variants are more prevalent in populations historically exposed to low intake of preformed LCPUFAs, such as ancestral Asian populations; in contrast, less efficient FADS variants are more common in populations consuming LCPUFAs-rich diets, such as European and North American populations. Accordingly, DHA metabolism is not uniform but reflects a dynamic interplay between genetic background and dietary intake [12,13,14]. This gene–diet interaction contributes to variability in DHA status and may partly explain the heterogeneity observed in clinical outcomes across studies.

In addition to these factors, Δ-6 desaturase activity is reduced in conditions such as chronic alcoholism and in diabetes, whereas Δ-5 desaturase activity may be impaired by low availability of vitamin C, niacin and zinc. Although high ALA levels in diet can increase DHA endogenous synthesis, it is not sufficient to achieve optimal levels: to increase DHA levels in the body, dietary supplementation of DHA is the most effective strategy [15]. In particular, during periods of rapid growth—such as the third trimester of pregnancy and early infancy—the endogenous synthesis of LCPUFAs is generally insufficient to meet the high demands of the developing brain and retina. Consequently, an adequate maternal dietary intake and placental transfer of DHA are essential to ensure optimal fetal accretion.

DHA bioavailability is strongly influenced by its digestive behavior, molecular form, and delivery system. Moreover, DHA metabolism is characterized by a balance between functional incorporation into neural membranes and susceptibility to oxidative degradation. In fact, DHA is particularly prone to lipid peroxidation, which may reduce its bioavailability and functional impact, especially under conditions of oxidative stress. In the brain, DHA turnover is tightly regulated, and its incorporation from plasma reflects ongoing metabolic consumption and signaling activity, supporting its role as a dynamic component of neurotransmission and membrane remodeling [16,17].

DHA confers membrane fluidity and influences the electrical, chemical, hormonal, and antigenic signals of the cells. Brain and photoreceptor cell membranes contain membrane phospholipids that are enriched in PUFAs. DHA accumulates in the brain through the major facilitator superfamily domain containing 2A (MFSD2A), a sodium-dependent lysophosphatidylcholine transporter expressed at the blood–brain barrier endothelium [18]. About 35% of total fats in the brain are PUFAs: they are critical for normal brain development and functioning [19]. Emerging evidence suggests that genetic variability in transport and metabolic pathways may influence DHA brain uptake. Although data on MFSD2A polymorphisms in humans remain limited, emerging evidence suggests that metabolic conditions such as maternal obesity may affect placental lipid transport mechanisms, potentially altering DHA availability to the fetal brain.

Beyond its structural role in neuronal membranes, DHA exerts neurodevelopment effects through membrane-dependent signaling and epigenetic regulation. DHA promotes the synthesis of bioactive mediators, which contribute to neurogenesis, synaptic plasticity and anti-inflammatory responses within the developing brain [16]. These processes occur within a highly dynamic epigenetic framework, where DNA methylation, chromatin modifications, and non-coding RNAs regulate gene expression during critical periods of brain development [20].

DHA is a dominant fatty acid of retinal phospholipids and affects rhodopsin content at discs, as well as photoresponses: to be included in retinal phospholipids, DHA is first activated to DHA-CoA and then it is esterified, obtaining phosphatidic acid (PA) by lysophosphatidic acid acyltransferase 3 (LPAAT3). LPAAT3 is expressed in DHA-rich tissues, such as retinas, which contain high amounts of DHA-containing phospholipids (PL-DHA). Experimental studies in animal models assert LPAAT3 deficiency dramatically lowers PL-DHA levels in the outer segment (OS) of photoreceptors and impairs visual functions [21]. In fact, examining the organization of rhodopsin and the structure of photoreceptor cell membranes in mice, it was seen DHA deficiency can impair vision due to photoreceptor cell dysfunction, which is caused by reduced activity of rhodopsin [22]. These findings provide a biological basis for clinical observations linking DHA status to early visual development, suggesting that adequate DHA availability during critical periods may be essential for optimal visual function in infancy.

Experimental *n*-3 LCPUFA deficiency during pregnancy and lactation in animal models is associated with adverse outcomes in offspring, including impairments in visual function, learning, memory, and motor development. Conversely, maternal DHA supplementation during pregnancy has been shown to increase brain DHA content in the offspring and to improve neurodevelopmental outcomes in mice [23,24].

DHA, ARA and EPA are substrates for the biosynthesis of eicosanoids, including prostaglandins, thromboxanes and leukotrienes via the cyclooxygenase (COX) and lipoxygenase (LOX) pathways. These lipid mediators regulate vascular tone, inflammatory responses, and cellular signaling pathways. Beyond this role, experimental studies have demonstrated that DHA directly promotes the activation of membrane-associated G-protein-coupled receptor (GPR) 120 and GPR 40 activation and anti-inflammatory signaling. DHA downregulates the pyrin domain-containing 3 (NLRP3) inflammasome and activates peroxisome proliferator-activated receptors (PPAR) upregulating PPAR-targeted genes, improving insulin sensitivity, and reducing plasma triglyceride levels and inflammation [25,26].

DHA and *n*-3 LCPUFA are not only constituents of lipid bilayer membrane, but they are also metabolized in specific bioactive mediators such as docosanoids and elovanoids, which play a key role in the regulation of cellular homeostasis [27,28].

These anti-inflammatory pathways are particularly relevant in early life, where dysregulated inflammatory responses contribute to major neonatal morbidities such as necrotizing enterocolitis and bronchopulmonary dysplasia.

Collectively, these biochemical and physiological properties underpin the critical role of DHA in brain development, visual function, immune regulation, and metabolic homeostasis during early life.

## 4. DHA: Influence During the First 1000 Days

DHA is considered crucial throughout the lifespan, particularly during pregnancy and breastfeeding, as it plays a fundamental role in neurodevelopment, visual development, and in establishing the foundations for cognitive and behavioral functions [2]. It is central to several pathophysiological mechanisms underlying immune function, visual acuity development, and neurodevelopment in newborns [29,30].

Both DHA and ARA are selectively transported across the placenta. Placental transfer of LCPUFA occurs predominantly during the third trimester, and brain accumulation is considerable. Maternal DHA, omega-3, and overall PUFA status during pregnancy is further influenced by multiple sociodemographic, lifestyle, dietary, and genetic factors, including pre-pregnancy BMI, diabetes, hypertensive disorders of pregnancy, interpregnancy interval, primiparity, and gestational weight gain. These factors act synergistically, influencing maternal DHA status through complex metabolic and physiological pathways. For example, dietary intake, genetic variability, and metabolic conditions such as obesity or gestational diabetes may collectively affect DHA synthesis and placental transfer. Notably, some of these factors, particularly maternal obesity and gestational diabetes mellitus (GDM) have also been shown to influence the efficacy of DHA supplementation, as discussed in subsequent sections. Maternal DHA concentrations increase across gestation, although the relative proportion of DHA among total lipids declines due to preferential placental transfer to the fetus. Consequently, the fetus—particularly during the third trimester—is highly dependent on adequate maternal DHA stores. When this phase is disrupted (e.g., late or very preterm birth, gestational diabetes mellitus, obesity, low-DHA diet), the newborn begins life at a disadvantage and should be supported with appropriate nutrition [31]. In preterm infants, gestational age (GA) correlates directly with ARA and DHA levels during the first week of life; those born at the lowest gestational ages are at highest risk of essential fatty acid deficiency [1,32,33,34].

Sinclair et al. identified three notable examples indicative of *n*-3 PUFA insufficiency, all pertaining to newborn populations. These included: (1) elevated 22:5*n*-6/DHA ratios—an established biomarker of DHA deficiency, reflecting a compensatory increase in *n*-6 docosapentaenoic acid (DPA*n*-6, 22:5*n*-6) when DHA availability is insufficient—in the cord arterial tissue of infants born to Hindu vegetarian women residing in London (UK); (2) elevated 22:5*n*-6/DHA ratios in newborns fed low-*n*-3 PUFA infant formulas in the UK and Australia; (3) extremely low DHA content in breast milk samples collected from women in northern Sudan, attributed to markedly unbalanced maternal diets. Together, these findings highlight the vulnerability of neonates to maternal dietary *n*-3 PUFA inadequacy and the critical importance of sufficient DHA intake during pregnancy and lactation [35].

Basak et al. conducted a review highlighting the central role of maternal PUFAs and the *n*-3/*n*-6 balance in shaping placental epigenetic regulation and fetal brain development. The authors describe how *n*-3 PUFAs influence angiogenesis, uteroplacental architecture, and DNA methylation processes. In contrast, diets low in *n*-3 and disproportionately high in *n*-6 fatty acids are associated with adverse placental epigenetic modifications, reduced placental transfer of long-chain PUFAs to the fetus, and increased risks of intrauterine growth restriction (IUGR), preeclampsia, and unfavorable neurodevelopmental outcomes [36].

Dietary patterns and maternal characteristics during pregnancy and lactation appear to be important modifiers of DHA-related neurodevelopmental outcomes, growth and adiposity indices, risk of allergy, asthma and eczema [37]. In the last decade, a substantial body of observational studies, randomized controlled trials (RCTs), and systematic reviews has investigated the role of maternal and early-life DHA exposure on offspring neurodevelopment, yielding heterogeneous and sometimes conflicting results.

For example, a recent secondary analysis of the PANDA study investigates the role of maternal neuroprotective nutrients such as DHA, choline, and carotenoids in infant brain function during early postnatal life, supporting a synergistic effect and underscoring the importance of adequate maternal intake of neuroprotective nutrients during pregnancy [38].

**Figure 1:**
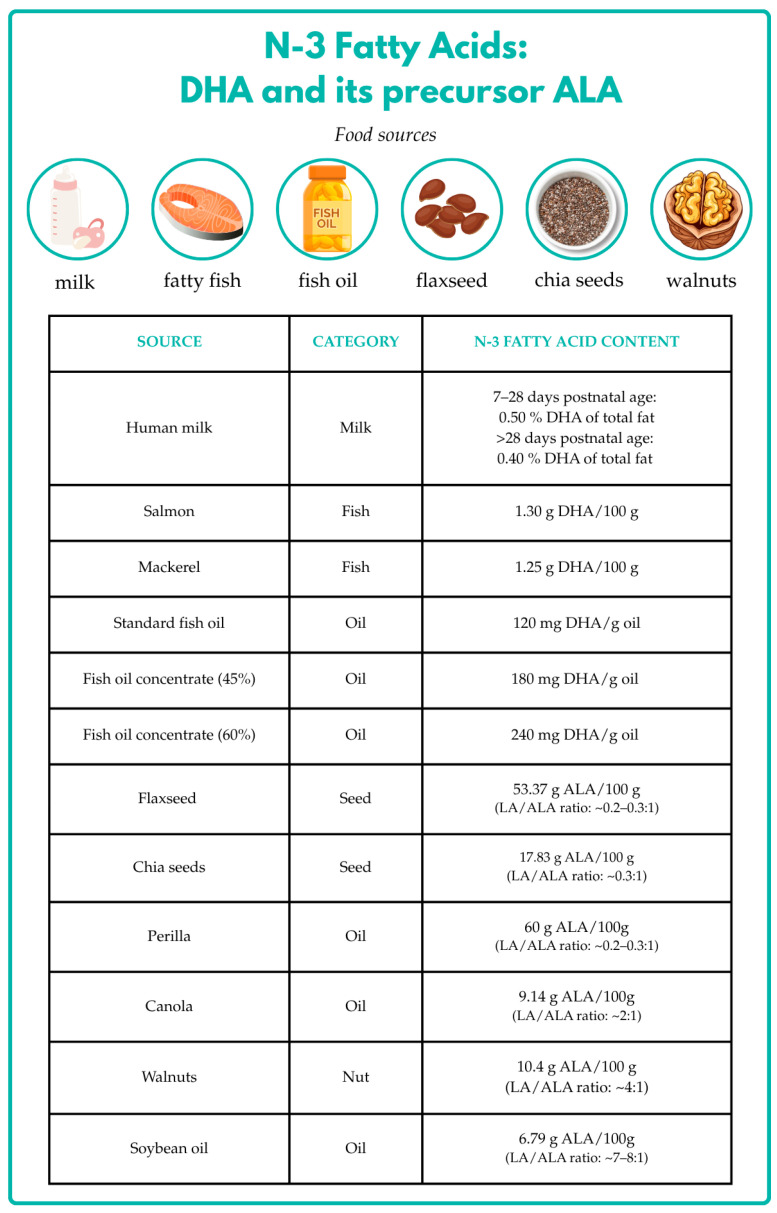
*N*-3 Fatty Acids: DHA and its precursor ALA food sources [6,39,40,41].

Major dietary sources of DHA include seafood and high-fat fish, whereas plant-based foods such as chia seeds, flaxseed, and walnuts provide α-linolenic acid (ALA), a precursor of DHA. Additional sources of ALA include vegetable oils such as canola, soybean, and perilla oil. The balance between LA and ALA intake is critical, as these fatty acids compete for the same enzymatic pathways, influencing endogenous DHA synthesis (Figure 1).

**Figure 2:**
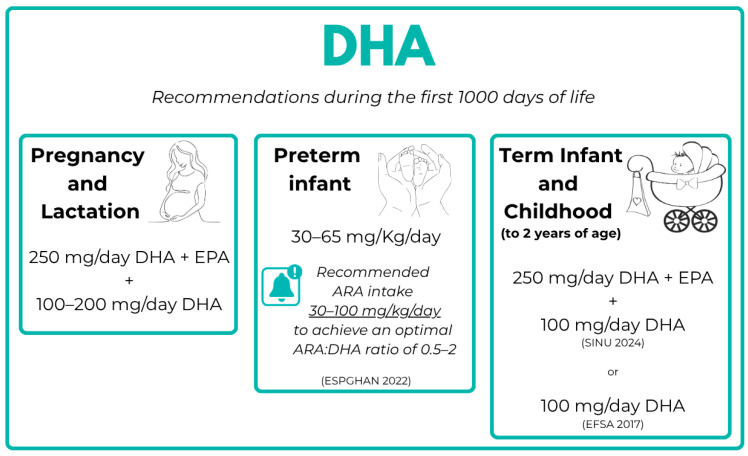
DHA recommendations during the first 1000 days of life [40,41,65,66,67].

Nutrition during the first 1000 days of life is considered one of the most influential determinants of child development. Within this framework, DHA intake recommendations during this critical window vary across developmental stages, as shown in Figure 2.

As a key structural component of the human brain and retina, DHA is essential for adequate brain formation, synaptogenesis, neuronal myelination, visual and prefrontal cortex development, and cognitive performance, and it also has a neuroprotective role [42,43]. DHA has been identified as critical for neurodevelopment during the first 1000 days, accumulating in neural tissues from the third trimester through the first 24 months of life. Increasing evidence in the literature indicates that sufficient concentrations of DHA are required from the fetal period through to early childhood to support optimal neurological development [44]. Inadequate nutritional intake during this window and omega-3 deficiency are associated with poorer developmental outcomes, mood disorders, attention deficits and impaired concentration, even when nutritional status is later restored [45,46,47,48]. Brain LCPUFAs levels in the offspring much depend on maternal PUFAs intake. Adequate DHA supply during the perinatal period is critical for optimal central nervous system development and function. Conversely, dietary *n*-3 LCPUFAs deficiency has been shown to impair neuronal plasticity. Beluska-Turkan et al. highlighted nutritional and knowledge gaps regarding nutrition and supplementation for the first 1000 days, emphasizing that omega-3 fatty acids are required for brain and eye development [49]. Public health policies should underline access to quality food for preconceptional, pregnant, and lactating women [50,51].

Basak et al. pointed out that low maternal DHA levels are associated with an increased risk of cognitive and behavioral impairments in offspring, and recommended a maternal intake of at least 250–500 mg/day of *n*-3 LCPUFAs to support optimal fetal development. If the mother reaches late pregnancy with low DHA stores, especially in high-risk pregnancies, the infant may start life with a neurodevelopmental disadvantage [52]. Similarly, Dervarshi et al. reported that both gestational diabetes mellitus (GDM) and preeclampsia are associated with altered maternal omega-3 status, disruptions in placental *n*-3 LCPUFA metabolism, and reduced cord blood *n*-3 LCPUFA concentrations, perturbations which may adversely influence infant neurodevelopment and contribute to impaired brain health later in life [53].

In a review examining the roles of DHA and ARA during pregnancy and lactation, with a particular focus on gestational obesity, Muñoz et al. reaffirmed the importance of these long-chain PUFAs for visual and cognitive development as well as for early growth. The authors highlight that maternal obesity and diets characterized by an elevated *n*-6/*n*-3 PUFA ratio can adversely modify both placental and breast milk lipid profiles, with downstream effects on breastfeeding duration and child growth trajectories. Evidence also indicates that *n*-3 PUFA supplementation, such as fish oil, in obese pregnant women may improve offspring cognitive outcomes and maternal–infant inflammatory profiles [54].

DHA is also a structural component of immune cell membranes, exerting immunomodulatory effects through multiple, interrelated biological pathways [55]. DHA exhibits antioxidant, anti-inflammatory, antiangiogenic, and antiproliferative properties [56,57,58].

Growing evidence suggests that maternal supplementation with *n*-3 long-chain polyunsaturated fatty acids (LCPUFAs) during pregnancy and breastfeeding may reduce the risk of allergic disease in offspring (e.g., eczema in children), likely through the anti-inflammatory properties of *n*-3 LCPUFAs [59,60]. Richard et al. conducted a systematic review highlighting the role of DHA in immune system development. Evidence from nutritional intervention studies indicates that maternal fish oil supplementation during late pregnancy and/or lactation, as well as the use of infant formulas enriched with DHA and ARA, can modulate immune biomarkers and support the establishment of oral tolerance during the first year of life [61].

Although both *n*-6 and *n*-3 LCPUFAs are required for normal cardiac development, excessive *n*-6 intake promotes pro-inflammatory and pro-thrombotic pathways and may disrupt fetal cardiac endocannabinoid signaling, thereby potentially increasing long-term cardiovascular risk [62].

It was also seen that alcohol reduces maternal–fetal DHA levels but, simultaneously, *n*-3 PUFA and especially DHA can have protective role as partial neuroprotection mitigating FASD-related effects (oxidative stress, inflammation, synaptic plasticity) [63,64].

### 4.1. DHA During Pregnancy and Lactation

Maternal DHA status has emerged as a critical determinant of fetal neurodevelopment, influencing not only brain maturation but also birth weight, early growth trajectories, and the long-term risk of obesity and metabolic dysfunction in offspring.

Maternal diet, body weight, and lifestyle factors may significantly influence infant immune function, organ development, and metabolic programming during the first 1000 days of life [68]. The effect of *n*-3 LCPUFAs supplementation—including dosage—in healthy pregnant women, on birth weight and subsequent childhood weight, have been extensively investigated.

A dietary source of DHA is required during pregnancy and lactation to include the needs of both the mother and the infant. According to current nutritional guidelines, DHA requirements increase by approximately 100–200 mg/day during pregnancy, in addition to the baseline recommended intake of 250 mg/day of combined EPA and DHA, thus referring to total daily intake rather than supplemental dose alone. To meet these needs, women of childbearing age are advised to consume one to two servings of fish per week, preferably including fatty fish (e.g., mackerel, salmon, eel, herring) or semi-fat fish (e.g., mullet, carp, sardines), which are naturally rich in DHA. When habitual fish consumption is low or absent, direct DHA supplementation of 100–200 mg/day is recommended, including during the puerperium, in line with national dietary guidelines (SINU) [65,66,67].

Massari et al. conducted a randomized controlled trial in which 176 pregnant women were allocated to receive either a multiple micronutrient supplement (MMS) plus DHA or no supplementation. Participants in the intervention group received a daily soft-gel capsule containing 12 vitamins, six minerals, and 200 mg of DHA, designed to meet nutritional requirements during pregnancy. Supplementation was initiated between 13 and 15 weeks of gestational age and continued until delivery. The intervention complemented habitual dietary intake and resulted in a significant improvement in maternal DHA status. However, the study lacked a placebo group and was conducted in an unblinded manner [69].

Additional insights are provided by studies on dietary sources of DHA. Khor et al. evaluated DHA content in breast milk, which directly reflects maternal diet. Their findings support recommendations for balanced PUFA intake during pregnancy and lactation to promote optimal fetal and childhood growth and development. They further noted that reduced maternal fish consumption can lead to an altered *n*-6/*n*-3 ratio, with potential consequences for infant brain development [70]. In line with these findings, Hibbeln et al. reported in their systematic review that maternal fish consumption during pregnancy is consistently associated with improved neurocognitive outcomes in offspring, supporting the role of dietary sources of *n*-3 LCPUFAs in early brain development [71].

Nevins et al. examined the impact of *n*-3 LCPUFA supplementation during pregnancy and/or lactation on child neurodevelopment in a systematic review addressing domains such as language, motor skills, socio-emotional functioning, and neuropsychiatric outcomes. They concluded that evidence remains insufficient and inconsistent, largely due to limited data on supplementation during lactation alone or combined pregnancy-plus-lactation interventions. While omega-3 intake during pregnancy may confer modest cognitive advantages, the authors emphasized that DHA is not a ‘smart pill’ and neurodevelopment ultimately requires structured follow-up, adequate caregiving environments, and enrichment beyond nutritional interventions [72].

During the pregnancy, several factors influence the onset of preeclampsia or preterm delivery: there are many strategies that can be implemented to reduce the risk. Among these, *n*-3 PUFAs supplementation has been studied, but evidence regarding the effectiveness of it in preventing preterm delivery or preeclampsia is mixed: many studies have shown a positive association between maternal *n*-3 PUFAs status during pregnancy and reduced risks of preeclampsia, preterm births and low birth weight [53,73,74,75,76,77,78,79,80,81,82,83], whereas others report no reduction in preterm birth rates following supplementation [84]. Best et al. summarized in the ISSFAL statement the available evidence pertaining to the effect of dietary omega-3 LCPUFA during pregnancy on preterm birth, evidencing that using perinatal commercially available products containing the recommended DHA level of 200 mg/day may not be sufficient to elevate maternal, placental, or fetal DHA concentrations compared to non-supplemented levels in normal-weight women. In contrast, in high-BMI women, supplementing DHA 200 mg/day was sufficient to increase DHA status in the maternal and fetal RBCs [85]. This finding may reflect differences in baseline DHA status, lipid metabolism, and distribution volume. Women with a higher BMI may have lower baseline DHA levels or altered lipid turnover, making them more responsive to supplementation at standard doses. Conversely, normal-weight women with a relatively higher baseline DHA status may require higher intakes to achieve measurable increases. In this context, baseline nutritional status and metabolic conditions should be carefully considered when evaluating the efficacy of DHA supplementation.

Maternal *n*-3 PUFA intake has also been shown to reduce offspring adiposity by modulating glucose and lipid metabolism and influencing fetal thermogenic development and skeletal growth dynamics [86].

Observational studies assessed associations between maternal or neonatal *n*-3 LCPUFAs or trans-fatty acid (TFA) and birth weight and weight during childhood. Evidence supports a positive relationship between maternal or neonatal *n*-3 LCPUFAs status and both birth weight and weight trajectories into childhood. This association was most apparent with higher-dose DHA and/or EPA supplementation, specifically at intakes of approximately 650 mg/day [87].

An observational study conducted in the Bogor district of Indonesia enrolled 142 women in their third trimester and followed them through delivery. The authors reported that low maternal intake of *n*-3 LCPUFAs, reflected in an elevated *n*-6/*n*-3 ratio, may contribute to the pathogenesis of several diseases. Higher maternal dietary DHA intake was associated with a lower infant fat mass at birth, whereas greater intake of total fat and *n*-6 PUFAs correlated with increased neonatal adiposity at specific anatomical sites. The increase in *n*-3 LCPUFAs intake during pregnancy is suggested to be confer benefits on offspring body composition [88]. Indeed, as discussed by Shrestha et al., the high intake of *n*-6 PUFAs typical of Western diets may influence fetal programming by impairing fetal growth and predisposing offspring to obesity and metabolic dysfunction later in life [62].

Meher et al. conducted a longitudinal study enrolling pregnant women and assessing maternal erythrocyte DHA across gestation. Maternal erythrocyte DHA at T1 (16–20 weeks’ gestation) was positively associated with infant birth weight. Women who delivered low-birth-weight (LBW) infants exhibited higher maternal erythrocyte total omega-6 LCPUFAs and ARA levels at T2 (26–30 weeks’ pregnancy) as well as lower erythrocyte *n*-3 LCPUFAs and higher *n*-6 and saturated fatty acid (SFA) levels at delivery [89].

A recent study investigated the associations between maternal and cord plasma fatty acid profiles at delivery, birth size, and child anthropometry at 3–7 years of age. Kadam et al. recruited a large Indian mother–child cohort. Mothers delivering small-for-gestational-age (SGA) infants exhibited lower total PUFAs, higher monounsaturated fatty acids (MUFAs), and an increased Δ6-desaturase index, while cord blood DHA and total *n*-3 PUFA levels were paradoxically higher in SGA infants. At follow-up, children born SGA showed persistently lower anthropometric measures, including BMI and circumferences. Importantly, a higher maternal *n*-6/*n*-3 ratio was positively associated with greater offspring adiposity, and cord Δ6-desaturase activity was independently associated with increased skinfold thickness. These findings suggest that maternal PUFA imbalance during pregnancy—particularly a higher *n*-6/*n*-3 ratio and altered desaturase activity—may influence fetal growth patterns and program adiposity risk in early childhood, underscoring the relevance of fatty acid quality during gestation [90].

The effects of maternal DHA and *n*-3 LCPUFAs status on offspring behavioral and cognitive outcomes have been extensively investigated. Overall, while biological plausibility and observational data consistently support a role for DHA in early brain development, randomized controlled trials have yielded heterogeneous and often inconsistent results, particularly for long-term cognitive and behavioral outcomes.

LCPUFAs have been implicated in neurodevelopmental disorders, with evidence suggesting that maternal PUFA imbalances, in particular, an elevated maternal *n*-6/*n*-3 ratio, has been associated with suboptimal neurodevelopmental outcomes and may increase the risk of autism spectrum disorder (ASD), attention-deficit/hyperactivity disorder (ADHD), and schizophrenia [62].

Ramakrishnan et al. conducted a randomized, placebo-controlled trial in which 1094 pregnant women were assigned to receive either 400 mg/day of DHA or placebo from 18–22 weeks’ gestation until delivery. Cognitive performance, behavioral functioning, and attentional outcomes were assessed in 797 offspring at 5 years of age using the McCarthy Scales of Children’s Abilities (MSCA), the parental scale of the Behavioral Assessment System for Children, Second Edition (BASC-2), and the Conners’ Kiddie Continuous Performance Test (K-CPT). The findings indicated that DHA supplementation during the second half of pregnancy may improve sustained attention in offspring beyond infancy, but limitations, such as the lack of direct measures of DHA status and the absence of detailed information on the quality of the child’s learning environment, could have influenced these results [91].

In a double-blind, randomized, placebo-controlled trial, Sass et al. investigated *n*-3 LCPUFAs supplementation during the third trimester of pregnancy within the COPSAC2010 mother–child cohort, which included 736 Danish women and their children. The primary outcome of the intervention was the incidence of recurrent asthma-like symptoms. Maternal *n*-3 LCPUFA supplementation was associated with earlier attainment of gross motor milestones in boys, enhanced cognitive performance at 2.5 years of age, improved early language development, and a reduced impact of emotional and behavioral difficulties at 6 years. The observed sex-specific differential responses to supplementation may reflect variations in essential fatty acid metabolism [92].

Tarui et al. explored the placenta–brain lipid axis, with particular emphasis on DHA, and its relevance to neurodevelopmental disorders such as ADHD and ASD. Their findings indicate that low maternal DHA status and maternal obesity are associated with increased risks of ADHD and ASD in offspring [93]. Because the placenta tightly regulates *n*-3 LCPUFA transfer to the fetus, maternal obesity and systemic inflammation may impair this process, resulting in suboptimal fetal DHA supply. Emerging evidence also suggests that *n*-3 supplementation during pregnancy may partially mitigate the adverse effects of maternal obesity on placental lipid metabolism [71].

However, evidence is still controversial: the impact of DHA interventions during the first 1000 days of life on child behavioral functioning was reviewed in 2021 by Gould et al., who examined RCTs, and found that prenatal or early postnatal DHA supplementation does not improve later child behavioral functioning and in some large trials, DHA supplementation reports worse outcomes (e.g., irritability, attention problems) [48].

DHA supplementation during pregnancy has been examined regarding its influence on brain maturation and visual development. Colombo et al. conducted a randomized trial in which pregnant women received either 800 mg or 200 mg of DHA daily, and their infants were later assessed using a visual habituation protocol augmented with simultaneous measurement of heart rate at 4 and 6 months of age. Visual habituation was evaluated by quantifying infants’ visual and cardiac responses to repeated stimulus presentations. Their findings suggest potential benefits of DHA doses higher than currently recommended levels, but this work is limited by sample attrition over time, which was exacerbated by the COVID-19 pandemic [94]. The importance of DHA in visual and cognitive pathways is further explored by Mun et al. in a review examining the combined roles of choline and DHA during pregnancy and lactation. They highlighted their synergistic contributions to brain and visual development, emphasizing that deficiencies in either nutrient may exacerbate neurovisual impairment [95].

Observational evidence consistently supports the biological plausibility of DHA as a critical determinant of early brain development. Recent trials provide mixed findings. While observational studies consistently support an association between maternal DHA status and neurodevelopmental outcomes, randomized controlled trials have not uniformly confirmed these findings. Khandelwal et al. in the DHANI protocol compared the effect of DHA supplementation (400 mg/day) versus placebo in pregnant women from ≤20 weeks of gestation to 6 months postpartum on neurodevelopment at 6 and 12 months, assessed using the Development Assessment Scale for Indian Infants (DASII). The findings indicated that prenatal DHA supplementation, when continued into the postnatal period, was associated with improved neurodevelopmental outcomes at 1 year of age [96]. These results suggest that sustained prenatal and postnatal DHA exposure may be beneficial in early infancy, particularly in nutritionally vulnerable populations.

However, Gustafson et al., comparing high-dose (800 mg/day) versus standard-dose (200 mg/day) prenatal DHA supplementation in a randomized, longitudinal, double-blind, single-center Phase III superiority trial, showed that high-dose prenatal DHA supplementation increased maternal–infant DHA equilibrium in a dose-dependent manner. This biochemical improvement was not associated with differences in fetal neurodevelopmental indices, such as fetal heart rate variability or fetal autonomic brain age scores at 32 and 36 weeks’ gestation. The COVID-19 pandemic and related shutdowns resulted in an attrition exceeding expectations, preventing the study from reaching the planned sample size for the fetal autonomic brain age score (fABAS) outcome [97]. Likewise, another randomized trial of prenatal *n*-3 LCPUFA supplementation, evaluating cognitive and behavioral outcomes at 12 years of age, similarly reported no significant long-term effects of the intervention [98]. Gawlik et al. reviewed 29 randomized controlled trials evaluating the effects of DHA supplementation during the first 1000 days of life on language development in children. Only four trials demonstrated a positive effect on a language outcome: three of them originated from the same trial, indicating that evidence of benefit is limited [99].

About the link between neurodevelopment and DHA-maternal status, Gulliot et al. assessed whether high-dose maternal DHA supplementation in breastfed, very preterm infants improves neurodevelopmental outcomes at 18–22 months’ corrected age (CA) in a follow-up of a randomized, double-blind, placebo-controlled multicenter trial. In the original trial, lactating mothers of infants born before 29 weeks’ gestation were enrolled and randomized within 72 h of delivery to receive either DHA-rich algal oil or placebo until 36 weeks’ postmenstrual age. Neurodevelopment was examined using the Bayley Scales of Infant and Toddler Development, Third Edition (Bayley-III). Maternal DHA supplementation did not result in overall improvements in neurodevelopmental outcomes; however, subgroup analyses suggested a potential benefit in language development among infants born before 27 weeks’ gestation [100].

Furthermore, Shahabi et al., using data from the longitudinal ECLIPSES cohort, examined maternal *n*-6/*n*-3 status in 336 mother–infant pairs and its association with early neurodevelopment. A higher *n*-6/*n*-3 ratio in the third trimester was significantly associated with poorer motor development on the BSID-III, including deficits in fine motor skills, whereas no significant associations were observed in the first trimester. These findings suggest that excessive omega-6 intake in late pregnancy may impair early motor development, underscoring the importance of correcting PUFAs imbalances before the third trimester [101]. Similarly, Herrera et al. examined the nutritional implications of PUFAs intake during pregnancy and the neonatal period, emphasizing the central role of DHA and ARA in brain and retinal development as well as overall growth. Based on available evidence, the authors recommended a maternal intake of ≥300 mg/day of DHA during pregnancy and 200 mg/day during lactation. They noted that the risk of DHA deficiency is heightened in individuals following restrictive diets, particularly vegetarian or vegan diets without supplementation, and highlighted the importance of assessing maternal PUFAs intake while considering potential oxidative stress risks associated with excessively high doses [25].

The effects of DHA on neurodevelopment appear domain-specific. While some studies report benefits in early attention and visual processing, evidence for improvements in global cognition, language, and behavioral outcomes remains inconsistent. This variability likely reflects heterogeneity across studies, including differences in baseline maternal DHA status, supplementation timing and duration, dosage, and population characteristics (e.g., nutritional status and maternal obesity). In addition, variability in outcome measures and follow-up duration further limits comparability.

Importantly, neurodevelopment is influenced by multiple non-nutritional factors—such as caregiving quality, socio-economic conditions, and early environmental stimulation—which are often not adequately controlled for, complicating the interpretation of intervention studies.

Regarding dosage, lower intakes (200–300 mg/day), consistent with current recommendations, are generally associated with limited or inconsistent benefits, whereas higher doses (≥400–800 mg/day) may confer modest improvements in early neurovisual and attentional outcomes.

Overall, current evidence does not support routine high-dose DHA supplementation during pregnancy to improve long-term neurodevelopmental outcomes, although potential benefits may exist in specific high-risk populations.

Emerging research is exploring multimodal nutritional strategies during pregnancy and their correlation on offspring.

Bragg et al. evaluated choline and DHA supplementation during pregnancy in low- and middle-income countries, reflecting growing interest in multimodal nutritional interventions that target DHA-related pathways [102].

The NUHEAL follow-up study by Azaryah et al. evaluated the long-term effects of maternal fish oil (FO) and/or 5-methyl-tetrahydrofolate (5-MTHF) supplementation throughout pregnancy on offspring brain functionality at 9.5–10 years of age. Participants were clustered into two groups: the first one included 33 children from the FO group (FO or FO + 5-MTHF), while the second one included 24 children from the No-FO group (5-MTHF alone or placebo). Functional magnetic resonance imaging (fMRI) acquisitions showed FO, but not 5-MTHF, supplementation during the second half of pregnancy is associated with decreased functional connectivity of children’s brain networks at 9.5–10 years of age. These findings suggest that maternal FO supplementation may modulate the maturation of resting-state brain networks and exert long-term effects on cognitive processing in offspring [103].

Both prenatal and early postnatal windows appear to be critical periods during which fatty acid exposure may shape immune development through long-lasting programming effects.

Rodríguez-Santana et al. conducted a double-blind RCT in which 46 pregnant women received either a fish oil-enriched drink (320 mg/day DHA + 72 mg/day EPA) or a control from 28 weeks’ gestation through 4 months postpartum. Maternal *n*-3 LC-PUFA supplementation was associated with higher anti-inflammatory cytokines (IL-4, IL-10 and IL-2), lower maternal IL-6 levels inversely correlated with plasma DHA, and reduced infant TNF-α at birth and 2.5 months, indicating a shift toward an anti-inflammatory immune profile in early life [104]. In the preterm population, Valentine et al. further showed that maternal DHA supplementation with 1000 mg/day DHA increases breast milk DHA content to levels approximating third trimester accretion, and this is associated with reduced inflammatory markers in both mothers and infants [105]. Importantly, randomized trials have demonstrated that maternal fish oil supplementation reduces T-helper 2 immune responses in offspring and lowers the risk of persistent wheezing and asthma, particularly among children born to mothers with low baseline *n*-3 status [106].

However, not all findings are uniform. In the Seychelles Child Development Study Nutrition Cohort 2, a large observational study in a population with habitually high fish consumption, the authors investigated associations between maternal and child fish intake, PUFA concentrations, and childhood asthma. Fish intake was assessed by questionnaire, while PUFA concentrations were measured in maternal serum at 28 weeks’ gestation and in cord blood at delivery. Childhood asthma at 7 years of age was assessed using the ISAAC questionnaire. High maternal fish consumption during pregnancy was not associated with asthma in offspring. In contrast, elevated cord blood DHA concentrations were linked to a higher asthma prevalence—an unexpected finding that warrants further exploration of underlying mechanisms [107].

These discrepancies may be explained by several factors: while randomized controlled trials support an immunomodulatory role for DHA, characterized by reduced inflammatory responses and lower risk of wheezing, observational findings highlight the complexity of immune programming and suggests that baseline dietary patterns, genetic susceptibility, and environmental context may modify the effects of DHA exposure.

The progressive increase of LCPUFAs in fetal blood and tissues relative to maternal levels has been described as “bio-magnification”. After birth, the post-natal accumulation of LCPUFAs in infant tissues depends largely on maternal transfer through breastmilk, which naturally contains DHA and ARA. Yalagala et al. investigated molecular form of DHA and its influence on DHA concentration in human milk and its transfer to the offspring. They concluded lysophosphatidylcholine-DHA was more effective than triacylglycerol-DHA in enriching milk DHA and it could be considered as a new strategy to increase milk DHA content and to potentially improve brain and retinal health in infants [108]. Formula-fed infants maintain overall LCPUFAs percentages through compensatory incorporation of *n*-6 LCPUFAs; however, this process is incomplete in formula-fed preterm infants [29,109].

DHA concentrations in breast milk are influenced by the dietary linoleic acid (LA) to α-linolenic acid (ALA) ratio, which is often altered in maternal obesity. Obese mothers typically exhibit an increased *n*-6/*n*-3 PUFA ratio and reduced levels of key *n*-3 LCPUFAs, including ALA, EPA, and DHA. These alterations may adversely affect infant immune maturation and neurodevelopment, highlighting the impact of maternal obesity on breast milk quality [110]. A randomized trial among French lactating women compared three groups supplemented through food products with ALA (enriched margarine and rapeseed oil) for 15 days to the control group consuming olive oil. While breastmilk DHA levels did not differ between groups, LA and ALA levels varied: the lowest LA-ALA ratio (5.5) in breastmilk was observed in the group receiving enriched margarine and rapeseed oil. In this group, breast milk DHA levels were approximately 0.60% of total fatty acids [111].

He et al. conducted a double-blind, randomized, placebo-controlled parallel trial to evaluate whether DHA supplementation during pregnancy increases DHA concentrations in colostrum among Chinese women, and to assess whether continued postpartum supplementation is required to sustain DHA levels throughout lactation. In this cohort, maternal supplementation with 100 mg/day of DHA beginning in the third trimester significantly elevated DHA concentrations in colostrum. However, this dosage appeared insufficient to maintain DHA levels beyond the colostrum period. Moreover, maternal DHA supplementation was associated with alterations in the gut microbiota, specifically Lactobacillus abundance at 42 days postpartum in both mothers and their infants [112]. Similarly, Ueno et al. investigated the role of maternal diet and DHA concentration in human breast milk among Japanese women, concluding that it may be influenced by diet [113].

See Table S1 in Supplementary Materials, which highlights key study findings about DHA status and its supplementation during pregnancy and lactation.

### 4.2. DHA and Preterm Population

Preterm infants are particularly vulnerable to DHA deficiency due to multiple factors, including the interruption of fetal DHA accretion that normally occurs during the third trimester of pregnancy. Although both maternal DHA supplementation and direct postnatal DHA administration to the infant have been implemented with some success, uncertainty remains regarding the optimal dosage and the most effective mode of DHA delivery [114].

Optimal early nutrition, ensuring sufficient intake of both macronutrients and micronutrients, is fundamental for normal brain development. Moreover, enhanced nutritional support during the first weeks after birth has the potential to improve long-term neurodevelopmental outcomes [115,116].

A Position Paper from the ESPGHAN Committee on Nutrition about Enteral Nutrition in Preterm Infants in 2022 recommends a DHA intake from 30 to 65 mg/kg/day, providing sufficient intake of ARA from 30 to 100 mg/kg/day to achieve an ARA:DHA ratio between 0.5 and 2, which is considered to be safe [40,41]. This ratio underscores that DHA supplementation in preterm infants should not be considered in isolation, but rather within the context of balanced LCPUFA provision.

Several trials consistently showed that enteral DHA supplementation is feasible, well tolerated, and effective in improving biochemical DHA status in preterm infants, although circulating levels often remain lower than those observed in term infants at discharge. Baack et al. conducted a double-blind RCT to assess the feasibility, tolerability, and efficacy of daily enteral DHA supplementation (50 mg/day) in conjunction with standard nutrition for preterm infants born between 24 and 34 weeks’ gestation. Supplementation began within the first week of life and blood fatty acid profiles were measured at baseline, at the attainment of full enteral feeding, and near discharge. As expected, preterm infants exhibited significantly lower baseline DHA levels compared with term peers. Baseline DHA status may represent a key determinant of response to supplementation. Infants with lower initial DHA levels may derive greater biochemical and potentially clinical benefit, whereas those with relatively higher baseline levels may show a more limited response. DHA-supplemented infants demonstrated a progressive rise in circulating DHA across the study period, whereas placebo-treated infants receiving standard neonatal nutrition showed no improvement. Nonetheless, despite supplementation, preterm infants maintained lower DHA concentrations at discharge than term infants. Overall, daily enteral DHA supplementation proved feasible, well tolerated, and effective in improving DHA status and mitigating deficiency in preterm infants [117]. In a randomized control trial, Frost et al. evaluated whether supplementing 30 very-low-birth-weight neonates in the first 8 weeks of life was sufficient to reduce declines as well as allow increases in whole blood concentrations. Supplementing 120 mg of DHA + ARA seemed to maintain a steady DHA status, while blood DHA concentrations increased in infants receiving 360 mg of DHA + ARA. These differences persisted through 8 weeks of age, with significantly higher DHA and ARA levels in the LCPUFA-360 group. The supplement was generally well tolerated across all dosing groups, indicating that higher-dose LCPUFA supplementation is both feasible and effective in improving fatty acid status in very-low-birth-weight neonates [118]. Marc et al. in their meta-analysis examined two trials including infants born preterm before 29 weeks’ gestation who received high-dose DHA enteral supplementation to achieve 40 mg/kg/day compared with a control group receiving no or low-dose DHA. They found high-dose DHA in this specific population is not associated with an increased risk of severe BPD [119].

Early enteral feeding likewise plays a critical role, as it can influence major clinical endpoints—including necrotizing enterocolitis (NEC) and late-onset infection—by promoting functional maturation of the gastrointestinal tract and shaping microbial colonization patterns that might otherwise predispose to disease.

Several randomized controlled trials have explored the role of DHA supplementation in preventing NEC and modulating inflammatory responses in preterm infants. Bernabe-García et al. in 2021 demonstrated that the enteral administration of 75 mg/kg/day of DHA starting at the first enteral feed prevents NEC in preterm infants [120]. Similarly, Abou El Fadl et al. enrolled a total of 67 neonates, with gestational age equal or less than 32 weeks at birth and weight equal or less than 1500 g, in a prospective randomized controlled study, to evaluate the impact of DHA supplementation on proinflammatory cytokines release and the development of NEC in preterm neonates. Neonates were randomly assigned to DHA group or the control group. Modified Bell’s staging criteria for NEC were used for diagnosis and staging of NEC. Levels of Interleukin 1 beta (IL-1b) were measured at baseline and after 10 days. This study suggests that enteral DHA supplementation could have benefits in reducing NEC incidence in preterm neonates because of its immunoregulatory effect modulating transcription of regulatory cytokines. There are two important limits: the study was conducted in a single neonatal intensive care unit (NICU); only small blood volumes could be safely and ethically obtained from preterm infants to perform biochemical analyses. [121]. However, systematic evidence indicates that DHA supplementation alone may increase NEC risk if not combined with ARA, highlighting the importance of maintaining an appropriate DHA/ARA balance. Alshaikh et al. conducted a systematic review examining whether supplementation with DHA alone may increase the risk of NEC, evaluating the incidence of NEC with placebo-controlled effects, concluding that the addition of ARA is essential both to achieve the intended benefits of DHA and to avoid potential adverse consequences associated with insufficient ARA intake. Accordingly, concurrent ARA supplementation should be considered when introducing DHA into the diets of preterm infants [122].

Importantly, available evidence suggests that the effects of DHA on NEC may differ depending on whether it is administered alone or in combination with ARA. While some studies indicate that DHA supplementation may reduce NEC incidence through anti-inflammatory mechanisms, other evidence suggests that DHA administered without adequate ARA may increase NEC risk. This highlights the importance of maintaining a physiological DHA/ARA balance, rather than considering DHA supplementation in isolation.

In addition, postnatal undernutrition—particularly insufficient energy intake during the first 4 weeks of life—is an independent predictor of chronic lung disease. These findings underscore the need for vigilant monitoring and proactive optimization of early nutritional support in extremely preterm infants who are at high risk for developing bronchopulmonary dysplasia (BPD) [123]. Likewise, in the secondary analysis of data from the ImNuT (Immature, Nutrition Therapy) study, a randomized double-blind clinical trial, Wendel et al. concluded that administrating ARA and DHA to preterm infants was safe and could have beneficial effects on respiratory outcomes [124]. Nevertheless, clinical trials evaluating DHA supplementation for BPD prevention have yielded mixed results. For example, Marc et al. conducted a placebo-controlled RCT enrolling lactating women who delivered before 29 weeks of gestation within 72 h of delivery from 16 Canadian neonatal intensive care units. They found that high-dose maternal DHA supplementation (1.2 g/day) from delivery to 36 weeks’ PMA did not significantly improve BPD-free survival in breastfed infants born before 29 weeks’ gestation, although interpretation was limited by early trial termination [125]. This finding should be interpreted in light of earlier concerns raised by trials such as N3RO. The N3RO multicenter RCT enrolled 1273 infants born <29 weeks’ gestation from 13 neonatal centers in Australia, New Zealand and Singapore, and randomized them within 3 days of their first enteral feed to receive either an enteral emulsion providing 60 mg/kg/day of DHA or a control soybean-oil emulsion until 36 weeks’ postmenstrual age, concluding there was no lower incidence of BPD in the treated group than that in the control [126]. The influence of DHA on the development of BPD has also been studied in association or not with ARA: Dang et al. in their meta-analysis concluded enteral supplementation of DHA with or without ARA does not protect against the onset of preterm complications and increase the risk of BPD [127]. The impact of DHA supplementation on BPD remains controversial. Differences between earlier trials and more recent meta-analyses likely reflect heterogeneity in study populations, DHA dose and formulation, ARA co-supplementation, and outcome definitions. While some studies reported no benefit, others suggested a potential increased risk, particularly when DHA was administered without adequate ARA. This may reflect an imbalance in *n*-3/*n*-6 fatty acids, potentially interfering with the physiological inflammatory signaling required for lung development.

DHA supplementation has been investigated for other prematurity-related complications, including retinopathy of prematurity (ROP). Pivodic et al. studied DHA supplementation as a therapeutic strategy for preventing severe retinopathy of prematurity (ROP) [128]. Supplementation showed a protective effect compared to no supplementation; the authors emphasized the need for confirmation in larger cohorts before definitive conclusions can be drawn. Similar results have been shown in this double-blind parallel clinical trial, which highlighted enteral DHA supplementation can prevent stage 3 ROP and can reduce the risk of severe ROP [129]. Hellström et al. further highlighted that very preterm infants are at substantial risk of postnatal growth failure, a known contributor to the development of ROP. Optimizing nutritional intake could potentially promote growth and reduce ROP as a key preventive approach. Enteral supplementation with both ARA and DHA appears to be a simple intervention with the potential to reduce the incidence of ROP in this vulnerable population [130].

Neurodevelopmental outcomes in preterm infants have been investigated across multiple domains, including structural brain maturation, early cognitive performance, and long-term behavioral and executive functioning. However, findings remain heterogeneous, depending on the timing of supplementation, dosage, and duration of follow-up. Very preterm infants are particularly vulnerable to DHA deficiency due to the interruption of placental accretion during the third trimester, prompting multiple randomized trials evaluating postnatal DHA supplementation. However, evidence on long-term neurodevelopmental benefits remains heterogeneous. Some studies indicate that DHA supplementation may exert effects on structural brain maturation [31]. A double-blind, randomized controlled trial clustered infants born before 29 weeks’ gestational age to receive either ARA at 100 mg/kg and DHA at 50 mg/kg, or medium-chain triglyceride (MCT) supplement from the second day of life to 36 weeks’ postmenstrual age. The primary outcome of this study was aimed at evaluating brain maturation by diffusion tensor imaging (DTI) using Tract-Based Spatial Statistics (TBSS) analysis. This study suggests that supplementation with ARA and DHA at doses approximating in utero accretion rates enhances white matter maturation compared with control treatment, but conclusions are limited by small sample size and limited availability of primary outcome data, which can lead to generalizations, introducing potential selection bias [131]. Heath et al. studied PUFA and risk of neurodevelopmental deficits in preterm infants, showing that formulas containing 0.3% DHA + 0.6% ARA determine better neurodevelopmental outcomes than LCPUFA-free formulas [31].

Nonetheless, not all interventional studies have demonstrated neurodevelopmental benefits.

A follow-up study of the N3RO trial investigated the latency of distractibility during focused play at 18 months’ corrected age: results showed no differences between DHA and control groups in distractibility latency or related attentional measures. A critical limitation of this follow-up study is the potential selection bias: participating families differed from the original N3RO cohort and were predominantly of higher socio-economic status. In addition, Bayle-III scores were not powered to detect group differences [132]. Similarly, a 5-year follow-up study of very preterm infants who had received high-dose DHA in the neonatal period determined that high-dose postnatal DHA conferred no measurable benefits on behavioral functioning or executive abilities at 5 years of age [133]. These findings suggest that postnatal DHA supplementation alone may be insufficient to modify long-term neurobehavioral trajectories in very preterm infants, highlighting the importance of adequate fetal DHA exposure and the broader neonatal clinical context.

The effects of LCPUFAs on neurodevelopment have been investigated in synergy with choline, uridine-5′-monophosphate (UMP), cytidine-5′-monophosphate (CMP), and selected micronutrients essential for optimal brain development.

Andrew et al. in the Dolphin neonatal trial, a randomized, double-blind, placebo-controlled trial, valued as a primary outcome the cognitive composite score (CCS) of the Bayley Scales of Infant Development, Third Edition (Bayley-III), in neonates with neurodevelopmental impairment risk factors. They found not-improved neurodevelopmental outcomes in the group treated with nutrient powder formulated to be mixed with breast milk, infant formula, or food, containing DHA, EPA, ARA, choline, UMP, CMP, zinc, iodine, and vitamin B12 compared to the control group. However, interpretation is limited by several factors, including small sample size, heterogeneity in gestational age and brain injury etiology, and severity [134].

A follow-up study of the ImNuT trial, a randomized controlled trial enrolling 120 infants born before 29 weeks’ gestational age, was published by Gunnarsdottir et al. in 2025. They randomized infants into two groups: the first one received an enteral supplementation of both ARA 100 mg/kg/day and DHA 50 mg/kg/day, while the second received a medium-chain triglycerides supplementation from the second day of life until 36 weeks’ postmenstrual age (PMA). At 2 years’ corrected age (CA), neurodevelopment was evaluated using the Bayley Scales of Infant and Toddler Development, Third Edition (BSID-III), and the Peabody Developmental Motor Scales, Second Edition (PDMS-2). The follow-up analysis showed no significant differences between groups across BSID-III or PDMS-2 domains, indicating that ARA and DHA supplementation in very preterm infants did not confer measurable neurodevelopmental advantages at 2 years CA. It is important to underscore this follow-up study has been limited by the small sample size; in addition, language scores were unavailable in 17 children, excluded due to a non-Norwegian first language [135]. These findings are consistent with the broader pattern observed throughout this review: DHA supplementation in preterm infants appears to improve biochemical status more consistently than long-term neurodevelopmental outcomes, which remain heterogeneous across trials.

A study about RCTs involving preterm (<37 weeks’ gestation) or low-birth-weight (<2500 g) infants, commencing LCPUFA supplementation before term, and reporting neurodevelopmental outcomes, such as neurodevelopmental impairment (NDI), intelligence quotient (IQ), mental development index (MDI), Psychomotor Development Index (PDI), motor score, language scores and cognitive scores, included 3620 children from 13 randomized controlled trials. It suggests that while LCPUFA supplementation did not provide clear evidence of long-term improvements in IQ, it may reduce the risk of intellectual disability in preterm or low-birth-weight infants [136].

Gould J.F. et al. evaluated general intelligence at 5 years of age in children who had been enrolled in a randomized controlled trial of neonatal DHA supplementation aimed at preventing bronchopulmonary dysplasia. In the original trial, infants born before 29 weeks’ gestation were randomly assigned in a 1:1 ratio to receive either an enteral emulsion providing 60 mg/kg/day of DHA or a control emulsion, beginning within the first 3 days of enteral feeding and continuing until 36 weeks’ postmenstrual age or hospital discharge, whichever occurred first. For the follow-up study, children from 5 of the 13 participating centers were invited to undergo neurocognitive assessment at 5 years’ corrected age using the Wechsler Preschool and Primary Scale of Intelligence (WPPSI). The primary outcome was the full-scale intelligence quotient (FSIQ). Among enrolled participants, neonatal supplementation with the DHA emulsion was associated with modestly higher FSIQ scores at 5 years compared with control feeding, suggesting potential long-term cognitive benefits of early high-dose DHA exposure [137].

Shepherd et al. conducted a review aimed at evaluating the long-term neurodevelopmental and respiratory outcomes associated with DHA supplementation in infants born before 29 weeks’ gestation. Infants received either direct enteral DHA at ≥40 mg/kg/day or breast milk/formula containing ≥0.60% DHA of total fatty acids. Early cognitive advantages appear to be sustained; however, the risk of BPD remains a concern and requires ongoing long-term evaluation. Meta-analysis indicated enteral high-dose DHA in extremely preterm infants was not associated with differences in global cognition scores; however, higher scores were observed with the use of a direct emulsion [138].

Overall, while some studies suggest that DHA supplementation may support early brain maturation and short-term neurodevelopmental outcomes, evidence for sustained long-term cognitive and behavioral benefits remains limited and inconsistent.

Furthermore, in preterm infants, enteral PUFA intake has been shown to influence circulating oxylipin profiles, with DHA- and ARA-derived oxylipins inversely associated with precursor intake, indicating active regulation of the downstream lipid mediators involved in inflammatory and immune signaling [139]. These findings align with evidence that DHA, as a structural component of immune cell membranes, modulates immune function through coordinated effects on eicosanoid synthesis, T-helper cell polarization, and gene expression [140].

See Table S2 in Supplementary Materials, which highlights key study findings about DHA in preterm population and the relationship between DHA status and outcome in this population.

### 4.3. DHA During Infancy and Childhood to 2 Years of Age

The European Food Safety Authority (EFSA) recommend 100 mg/day of DHA in term infants and children to 2 years of age, while the Italian Society of Human Nutrition (SINU) recommends 250 mg/day of DHA + EPA plus 100 mg/day of DHA in infants to the 2nd birthday [65,66].

DHA accumulates in the brain from the third trimester of pregnancy and predominantly between birth and 2 years of age [141]. Several studies have explored the influence of DHA and *n*-3 LCPUFAs supplementation on early neurovisual outcomes across different developmental stages.

Tabilo et al. performed a systematic review of randomized controlled trials to examine the influence of *n*-3 PUFA supplementation on visual health across the lifespan. They reported generally more favorable effects in preschool- and school-aged children, particularly among those with ADHD. Overall, the evidence supports an important role of DHA in retinal maturation, and, despite some heterogeneity, ensuring adequate *n*-3 PUFAs intake appears advisable in high-risk settings such as prematurity or low dietary fish consumption [142].

Significant benefits of supplementation for infant cognitive development and visual acuity have been investigated by Shulkin et al., in a systematic review and meta-analysis about *n*-3 PUFAs supplementation and psychomotor and visual outcomes. Improvements were most pronounced for visual acuity, followed by gains in Bayley Scales of Infant Development mental and psychomotor development indices (MDI and PDI). No significant association emerged for overall childhood IQ; however, this finding should be interpreted with caution because of the limited number of studies available for the IQ analysis. The authors also identified potential benefits of *n*-3 PUFAs supplementation for visual acuity in both preterm and term infants and for MDI in preterm infants [143].

Despite these encouraging associations, the overall evidence remains inconclusive, and findings from studies evaluating postnatal DHA supplementation further highlight substantial variability.

Andrew et al., in the Dolphin double-blind RCT, randomized children between 1 to 18 months of age with suspected cerebral palsy receiving DHA, choline, and UMP supplementation for 2 years, and compared them with the control group who received no supplementation. The authors found no differences between the two groups for cognitive and language performance, as assessed by the cognitive composite score of the Bayley Scales of Infant and Toddler Development, Third Edition (CCS-Bayley-III), and the language composite score of the Bayley Scales of Infant and Toddler Development, Third Edition (LCS-Bayley-III), but they identified several limitations in interpreting the results, primarily related to the low number of recruited neonates, which was due to challenges in the early identification of cerebral palsy and the exclusion of some potentially eligible participants who had already received neonatal supplementation in a simultaneous trial conducted in the same geographic area. In addition, a higher withdrawal rate was observed in the treatment group [144].

Gawlik et al. reviewed 29 randomized controlled trials evaluating the effects of DHA supplementation during the first 1000 days of life on language development in children. Only four trials demonstrated a positive effect on a language outcome: three of them originated from the same trial, indicating that evidence of benefit is limited [99].

Hu et al. found mixed results: no statistically significant difference in Mental Development Index (MDI) scores was detected between infants receiving DHA supplementation and those given a placebo, while Psychomotor Development Index (PDI) scores were significantly higher among DHA-supplemented infants. Overall, BSID assessments indicate that DHA supplementation may offer modest neurodevelopmental benefits [145]. Nonetheless, the limited number of high-quality studies and methodological heterogeneity highlight the need for further research to clarify the specific roles of DHA supplementation in infants, pregnant women, and lactating mothers. Another study did not find significant association between DHA/EPA supplementation and any of the assessed cognitive parameters or birth weight [146].

Accumulating evidence indicates that early exposure to *n*-3 LCPUFAs is associated with more mature immune responses and a reduced risk of allergic and respiratory illness over the first years of life [55].

Clinical outcomes data support these immunological findings. Foiles et al. enrolled children from the Kansas City cohort of the DIAMOND (DHA Intake and Measurement of Neural Development) study, who were fed either a control formula without LCPUFA or one of three formulas with LCPUFA from DHA and ARA. A reduction in allergic diseases, upper respiratory infections, wheezing, and asthma in the first year of life, as well as a delay in the time to the first allergic illness and skin allergic illness, was reported when healthy, full-term infants were fed ARA- and DHA-supplemented formula [147].

However, evidence remains inconsistent: long-term follow-up studies further suggest that these protective effects may extend into adulthood, supporting the concept of fetal immune programming by maternal fatty acid intake [59].

See Table S3 in Supplementary Materials, which summarizes key study findings about DHA status and its supplementation during infancy and early childhood up to 2 years of age.

## 5. Discussion

**Figure 3:**
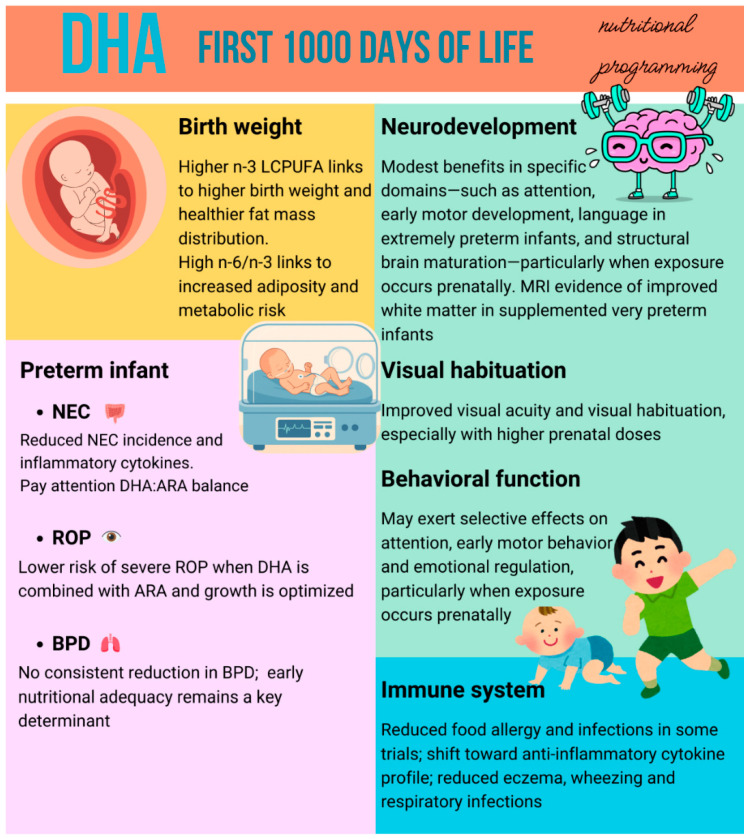
The key roles of DHA during the first 1000 days of life.

This review integrates physiological and clinical evidence supporting the biological importance of DHA across the first 1000 days of life (Figure 3). The biological effects of DHA during early life are strongly time-dependent: the third trimester of pregnancy is a critical window for placental transfer and fetal brain accretion. During late gestation, DHA delivery to the fetus increases markedly in parallel with neurogenesis, synaptogenesis, and neurovascular development. Consequently, maternal DHA depletion or disruption of placental transfer (e.g., in preterm birth) may irreversibly alter early neurodevelopmental trajectories.

Trials examined in this review consistently show that enteral DHA (often with ARA) is feasible and improves biochemical status in both mothers and infants. This represents one of the most robust and reproducible findings across trials. In contrast, clinical outcomes are considerably more heterogeneous. While some studies report benefits in specific neurodevelopmental domains, including sustained attention, early language acquisition, and early neurovisual processing, evidence for improvements in global cognition and long-term behavioral outcomes remains inconsistent. This discrepancy highlights a clear gap between biochemical efficacy and clinical effectiveness.

Although higher-dose prenatal DHA supplementation increases maternal–infant DHA equilibrium in a dose-dependent manner, this biochemical normalization does not consistently translate into measurable improvements in fetal autonomic or neurodevelopmental indices. These findings reinforce the concept that adequate DHA status is necessary but not sufficient to ensure optimal neurodevelopment and that timing, baseline status, and biological context are critical modifiers of response.

Emerging evidence further underscores that fatty acid balance, rather than DHA dose alone, is a critical determinant of biological and clinical outcomes. Pregnancy LCPUFAs metabolism contributes to growth and neurodevelopmental programming, influencing inflammatory and metabolic pathways. A higher maternal *n*-6/*n*-3 ratio in late pregnancy has been linked with poorer infant motor outcomes, as well as maternal LCPUFAs imbalance and FADS activity, which have been linked with later childhood adiposity. These data support a shift from a single-nutrient perspective toward a more integrated view of lipid quality and balance during gestation and lactation. Human milk is a complex biological system, in which nutrients and bioactive components act synergistically to support infant health. The functional properties of human milk reflect the combined influence of maternal diet, immediate environmental conditions, and evolutionary adaptations within the maternal–infant dyad. Within this system, milk fat globules play a central role: beyond energy supply, milk fat globules deliver the bioactive and signaling molecules involved in immune and developmental processes [148]. Moreover, synergism is emerging in studies combining DHA with choline, UMP, and micronutrients. Nonetheless, existing trials have not consistently shown superior neurodevelopmental outcomes compared with controls, indicating that formulation, dose, adherence, baseline status, and endpoint sensitivity remain critical.

In very preterm infants, early enteral DHA supplementation—often administered with ARA—is feasible, well tolerated and improves circulating DHA levels. However, long-term neurodevelopmental outcomes remain heterogeneous. These findings imply that prematurity-related comorbidities—including infection, inflammation, BPD, and ROP—may disguise nutritional effects, highlighting the need for targeted approaches.

Specifically for prematurity-related morbidities, the literature presents conflicting results. Randomized trials indicate that DHA supplementation may reduce NEC incidence and pro-inflammatory cytokine expression. However, systematic evidence raises doubt that the somministration of DHA alone may increase the risk of development of NEC if not accompanied by adequate ARA administration: DHA should not be considered in isolation in the preterm setting, as the DHA:ARA ratio appears crucial to intestinal integrity and immune signaling. The concept of fatty acid balance, particularly the DHA:ARA ratio, emerges as a central theme across both experimental and clinical evidence, representing a key take-home message of this review. For BPD and ROP, findings are largely inconsistent: some studies report no benefit or even potential harm, particularly at higher DHA doses. These discrepancies likely reflect differences in baseline DHA status, disease severity, and co-existing morbidities, which may obscure potential nutritional effects.

DHA also acts as a key modulator of immune development. As a structural component of immune cell membranes, DHA influences eicosanoid synthesis, T-helper cell polarization, and transcriptional regulation of inflammatory pathways shifting toward an anti-inflammatory cytokine profile. However, while some studies report reduced risk of allergic disease and respiratory illness, others—particularly in populations with high baseline DHA exposure—have shown paradoxical associations with increased asthma risk. These observations underscore that immune programming by DHA is context dependent.

Taken together, these findings suggest that DHA acts as a developmental modulator, with effects depending on timing, dose, fatty acid balance, baseline nutritional status, and clinical context. In addition, genetic polymorphisms in gene clusters involved in DHA metabolism indicate it is not uniform across individuals or population but reflects a dynamic interplay between genetic background, dietary intake, epigenetic regulation, and metabolic context. This variability may modulate the efficiency of endogenous DHA synthesis, contributing to interindividual differences in DHA status, and supports the need to move toward tailored nutritional strategies, in which DHA intake is adapted to individual metabolic capacity, dietary context, and population-specific characteristics in order to optimize early-life development. Future research is required to clarify the role of DHA during the first 1000 days of life to pick out strong guidelines and precision-based nutritional strategies.

DHA should not be interpreted as a single-target intervention, but rather as part of a broader nutritional and developmental framework in which timing, dosage, fatty acid balance, baseline status, and clinical context critically determine outcomes.

Overall, interpretations of the available evidence are constrained by small samples sizes, substantial attrition—frequently exacerbated by the COVID-19 pandemic—and, in some cases, early trial termination. Additional limitations include single-center designs, lack of placebo control or blinding, selection bias in follow-up cohorts and heterogeneity in gestational age and brain injury severity. Together, these factors underscore the need for larger, adequately powered, multicenter trials with rigorous methodology and comprehensive outcome evaluation. Studies should also be carried out in low- and middle-income countries (LMICs), in which DHA intake is not optimal because of limited access to DHA food sources, in order to draw robust conclusions regarding its contribution to healthy growth and neurodevelopment [149].

However, there are some trials still ongoing.

In 2024, Andrew et al. designed a protocol study of the DOLFIN trial: the primary outcome is the Parent Report of Children’s Abilities—Revised non-verbal cognitive scale at 24 months post-estimated date of delivery in infants born extremely preterm (EP, <28 weeks’ gestation) or infants > 35 weeks’ gestation who received hypothermia for hypoxic-ischemic encephalopathy (HIE). The treated group is receiving 1 mg/kg/day of the powered supplement (DHA, EPA, ARA, choline, UMP, and CMP, zinc, iodine, and vitamin B12) daily (maximum 12 g/day) and it is added to the milk feed when infants reach full enteral feeding [150].

In the Dolphin CONTINUE (Concept of Nutrition to Improve Neurodevelopment in Early Life) trial, Janson et al. recruited infants born at <30 weeks’ gestation and randomized them to either a supplementation group, receiving DHA, choline and UMP (1 g/kg/day, maximum 12 g/day), or a control group receiving a control product. The intervention was administered from term-equivalent age to 12 months corrected age (CA). The primary outcome parameter is white matter integrity at 3 months CA, assessed using diffusion tensor imaging (DTI) on MRI scanning, while secondary outcomes include other MRI parameters, product safety, and language, cognitive, motor, and behavioral development (assessed at 12 and 24 months CA, using the Bayley Scales of Infant Development III and digital questionnaires (Dutch version of the Communicative Development Inventories (N-CDI), Ages and Stages Questionnaire 4 (ASQ-4), and Parent Report of Children’s Abilities—Revised (PARCA-R)). The hypothesis is that supplementation can improve white matter integrity and subsequent neurodevelopmental outcomes in very preterm infants, but results are awaited to clarify the clinical relevance of this synergistic nutritional strategy [151].

## 6. Conclusions

DHA is biologically essential during the first 1000 days, with crucial roles in brain and retinal development as a structural component of neuronal and retinal membranes and immunomodulatory effects as a precursor of the lipid mediators that regulate inflammation and cellular homeostasis.

Supplementation consistently improves DHA status, but clinical benefits are heterogeneous and often appear in specific domains (attention, early language, psychomotor and visual outcomes) rather than global cognition. Importantly, genetic polymorphisms may modulate DHA metabolism, underscore the need for precision-based nutritional strategies in early life rather than uniform supplementation approaches. Moreover, DHA requirements should be considered within the broader context of essential fatty acid balance, as interactions among LA, ALA, and their long-chain derivatives influence competitive enzymatic pathways and downstream biological effects, such as inflammatory responses and neurodevelopmental outcomes.

Timing and context are critical: disruptions of late-gestation accretion (e.g., very preterm birth, placental dysfunction, maternal metabolic disease, obesity, low-DHA diets) determine unfavorable outcomes regarding neurodevelopment, birth weight and immune system development. In very preterm infants, enteral DHA (often with ARA) is feasible and improves biochemical status; findings from imaging studies suggest potential benefit from supplementing preterm infants with DHA and ARA and white matter maturation. For prematurity-related morbidities, DHA shows promising evidence for reduced incidence of NEC, but safety considerations highlight the importance of co-supplementation with ARA and balanced LCPUFA supply; evidence for BPD prevention is mixed, and ROP findings are encouraging but not definitive.

RCTs support a role for maternal and early-life *n*-3 LCPUFAs in promoting anti-inflammatory immune programming, impacting immune and allergic outcomes.

Nevertheless, translation into consistent clinical benefits—especially for long-term neurodevelopment—remains uncertain. Future research should prioritize DHA:ARA balance, focusing on integrated and context-specific strategies in order to optimize early-life nutritional programming. Future studies should standardize neurodevelopmental endpoints with long-term follow-up and high-risk phenotypes to define optimal dosing and target populations.

## Data Availability

No new data were created or analyzed in this study. Data sharing is not applicable to this article.

## Supplementary Materials

The following supporting information can be downloaded at: https://www.mdpi.com/article/10.3390/nu18081178/s1, Table S1: DHA during pregnancy and lactation, key study findings; Table S2: DHA in preterm population, key study findings; Table S3: DHA during infancy and early childhood (0–2 years), key study findings.

## References

[1] Panzeri C., Pecoraro L., Dianin A., Sboarina A., Arnone O.C., Piacentini G., Pietrobelli A. (2024). Potential Micronutrient Deficiencies in the First 1000 Days of Life: The Pediatrician on the Side of the Weakest. Curr. Obes. Rep..

[2] Weiser M.J., Butt C.M., Mohajeri M.H. (2016). Docosahexaenoic Acid and Cognition throughout the Lifespan. Nutrients.

[3] Sinclair A.J. (2019). Docosahexaenoic Acid and the Brain-What Is Its Role?.

[4] Wesołowska W., Bachoń E., Doligalska M., Stremel A., Leszyńska A., Linke J., Bałoniak Z., Kozłowska D., Bałoniak J., Tuszyńska W. (2025). Omega-3 Fatty Acids: Key Players in Cognitive Function and Brain Health. J. Educ. Health Sport.

[5] Basak S., Mallick R., Duttaroy A.K. (2020). Maternal Docosahexaenoic Acid Status during Pregnancy and Its Impact on Infant Neurodevelopment. Nutrients.

[6] Shahidi F., Ambigaipalan P. (2018). Omega-3 Polyunsaturated Fatty Acids and Their Health Benefits. Annu. Rev. Food Sci. Technol..

[7] Bhatt S., Kumari R., Deepika, Chopra R., Dhewa T., Kumari A. (2023). Omega-3 Fatty Acids: Nutritional Aspects and Their Role in Health and Diseases.

[8] Saini R.K., Prasad P., Sreedhar R.V., Naidu K.A., Shang X., Keum Y.S. (2021). Omega−3 Polyunsaturated Fatty Acids (PUFAs): Emerging Plant and Microbial Sources, Oxidative Stability, Bioavailability, and Health Benefits—A Review. Antioxidants.

[9] Coulson M., Mutch D.M. (2025). Molecular Insights into the Functional Roles of Variants in the FADS Gene Cluster on Omega-3 Long-Chain Polyunsaturated Fatty Acid Synthesis. Lifestyle Genom..

[10] He Z., Zhang R., Jiang F., Zhang H., Zhao A., Xu B., Jin L., Wang T., Jia W., Jia W. (2018). FADS1-FADS2 Genetic Polymorphisms Are Associated with Fatty Acid Metabolism through Changes in DNA Methylation and Gene Expression. Clin. Epigenetics.

[11] Kothapalli K.S.D., Ye K., Gadgil M.S., Carlson S.E., O’Brien K.O., Zhang J.Y., Park H.G., Ojukwu K., Zou J., Hyon S.S. (2016). Positive Selection on a Regulatory Insertion–Deletion Polymorphism in FADS2 Influences Apparent Endogenous Synthesis of Arachidonic Acid. Mol. Biol. Evol..

[12] Ihejirika S.A., Chiang A.H., Singh A., Stephen E., Chen H., Ye K. (2025). A Multi-Level Gene-Diet Interaction Analysis of Fish Oil and 14 Polyunsaturated Fatty Acid Traits Identifies the FADS and GPR12 Loci. Hum. Genet. Genom. Adv..

[13] Ye K., Gao F., Wang D., Bar-Yosef O., Keinan A. (2017). Dietary Adaptation of FADS Genes in Europe Varied across Time and Geography. Nat. Ecol. Evol..

[14] Li P., Zhao J., Kothapalli K.S.D., Li X., Li H., Han Y., Mi S., Zhao W., Li Q., Zhang H. (2018). A Regulatory Insertion-Deletion Polymorphism in the FADS Gene Cluster Influences PUFA and Lipid Profiles among Chinese Adults: A Population-Based Study. Am. J. Clin. Nutr..

[15] Ghasemi Fard S., Wang F., Sinclair A.J., Elliott G., Turchini G.M. (2019). How Does High DHA Fish Oil Affect Health? A Systematic Review of Evidence. Crit. Rev. Food Sci. Nutr..

[16] Kim H.Y., Huang B.X., Spector A.A. (2022). Molecular and Signaling Mechanisms for Docosahexaenoic Acid-Derived Neurodevelopment and Neuroprotection. Int. J. Mol. Sci..

[17] Jiang M., Hu Z., Chen X.D., Wu P. (2025). Unlocking the Release, Digestion and Absorption Kinetics of DHA from Different Fish Oil Delivery Systems: An In Vitro and Ex Vivo Study. Food Hydrocoll..

[18] Quek D.Q.Y., Nguyen L.N., Fan H., Silver D.L. (2016). Structural Insights into the Transport Mechanism of the Human Sodium-Dependent Lysophosphatidylcholine Transporter MFSD2A. J. Biol. Chem..

[19] Liu J.J., Green P., John Mann J., Rapoport S.I., Sublette M.E. (2015). Pathways of Polyunsaturated Fatty Acid Utilization: Implications for Brain Function in Neuropsychiatric Health and Disease. Brain Res..

[20] Peña C.J. (2026). Epigenetic Regulation of Brain Development, Plasticity, and Response to Early-Life Stress. Neuropsychopharmacology.

[21] Shindou H., Koso H., Sasaki J., Nakanishi H., Sagara H., Nakagawa K.M., Takahashi Y., Hishikawa D., Iizuka-Hishikawa Y., Tokumasu F. (2017). Docosahexaenoic Acid Preserves Visual Function by Maintaining Correct Disc Morphology in Retinal Photoreceptor Cells. J. Biol. Chem..

[22] Senapati S., Gragg M., Samuels I.S., Parmar V.M., Maeda A., Park P.S.H. (2018). Effect of Dietary Docosahexaenoic Acid on Rhodopsin Content and Packing in Photoreceptor Cell Membranes. Biochim. Biophys. Acta Biomembr..

[23] Dinel A.L., Rey C., Bonhomme C., Le Ruyet P., Joffre C., Layé S. (2016). Dairy Fat Blend Improves Brain DHA and Neuroplasticity and Regulates Corticosterone in Mice. Prostaglandins Leukot. Essent. Fat. Acids.

[24] Oguro A., Fujiyama T., Ishihara Y., Kataoka C., Yamamoto M., Eto K., Komohara Y., Imaoka S., Sakuragi T., Tsuji M. (2023). Maternal DHA Intake in Mice Increased DHA Metabolites in the Pup Brain and Ameliorated MeHg-Induced Behavioral Disorder. J. Lipid Res..

[25] Herrera E., Ortega-Senovilla H. (2023). Dietary Implications of Polyunsaturated Fatty Acids during Pregnancy and in Neonates. Life.

[26] Keelan J.A., Mas E., D’Vaz N., Dunstan J.A., Li S., Barden A.E., Mark P.J., Waddell B.J., Prescott S.L., Mori T.A. (2015). Effects of Maternal N-3 Fatty Acid Supplementation on Placental Cytokines, pro-Resolving Lipid Mediators and Their Precursors. Reproduction.

[27] Bazan N.G. (2018). Docosanoids and Elovanoids from Omega-3 Fatty Acids Are pro-Homeostatic Modulators of Inflammatory Responses, Cell Damage and Neuroprotection. Mol. Asp. Med..

[28] Christi W.W., Harwoo J.L. (2020). Oxidation of Polyunsaturated Fatty Acids to Produce Lipid Mediators. Essays Biochem..

[29] Lauritzen L., Brambilla P., Mazzocchi A., Harsløf L.B.S., Ciappolino V., Agostoni C. (2016). DHA Effects in Brain Development and Function. Nutrients.

[30] Mallick R., Basak S., Duttaroy A.K. (2019). Docosahexaenoic Acid,22:6n-3: Its Roles in the Structure and Function of the Brain. Int. J. Dev. Neurosci..

[31] Heath R.J., Klevebro S., Wood T.R. (2022). Maternal and Neonatal Polyunsaturated Fatty Acid Intake and Risk of Neurodevelopmental Impairment in Premature Infants. Int. J. Mol. Sci..

[32] Wilson N.A., Mantzioris E., Middleton P.T., Muhlhausler B.S. (2019). Gestational Age and Maternal Status of DHA and Other Polyunsaturated Fatty Acids in Pregnancy: A Systematic Review. Prostaglandins Leukot. Essent. Fat. Acids.

[33] Wilson N.A., Mantzioris E., Middleton P.F., Muhlhausler B.S. (2020). Influence of Sociodemographic, Lifestyle and Genetic Characteristics on Maternal DHA and Other Polyunsaturated Fatty Acid Status in Pregnancy: A Systematic Review. Prostaglandins Leukot. Essent. Fat. Acids.

[34] Wilson N.A., Mantzioris E., Middleton P.F., Muhlhausler B.S. (2020). Influence of Clinical Characteristics on Maternal DHA and Other Polyunsaturated Fatty Acid Status in Pregnancy: A Systematic Review. Prostaglandins Leukot. Essent. Fat. Acids.

[35] Sinclair A.J., Wang Y., Li D. (2023). What Is the Evidence for Dietary-Induced DHA Deficiency in Human Brains?. Nutrients.

[36] Basak S., Duttaroy A.K. (2023). Maternal PUFAs, Placental Epigenetics, and Their Relevance to Fetal Growth and Brain Development. Reprod. Sci..

[37] Mitguard S., Doucette O., Miklavcic J. (2023). Human Milk Polyunsaturated Fatty Acids Are Related to Neurodevelopmental, Anthropometric, and Allergic Outcomes in Early Life: A Systematic Review. J. Dev. Orig. Health Dis..

[38] Christifano D.N., Liao K., Mathis N.B., Carlson S.E., Colombo J., Chollet-Hinton L., Gustafson K.M. (2025). Neuroprotective Nutrients in Pregnancy and Infant Brain Function. Clin. Nutr. ESPEN.

[39] Makrides M., Adelaide S.A.J., Bhatia G., Bhatia J., Carlos Lifschitz G., Aires B., Adelaide S.A.E., Nel C., Town F.M., Ruemmele P. (2016). The Role of Docosahexaenoic Acid in the First 1000 Days.

[40] Lapillonne A. (2022). ESPGHAN Committee of Nutrition (CoN) Position Paper on Enteral Nutrition for Preterm Infants: Fat Supply.

[41] Embleton N.D., Jennifer Moltu S., Lapillonne A., Van Den Akker C.H.P., Carnielli V., Fusch C., Gerasimidis K., Van Goudoever J.B., Haiden N., Iacobelli S. (2023). Enteral Nutrition in Preterm Infants (2022): A Position Paper from the ESPGHAN Committee on Nutrition and Invited Experts. J. Pediatr. Gastroenterol. Nutr..

[42] Sambra V., Echeverria F., Valenzuela A., Chouinard-Watkins R., Valenzuela R. (2021). Docosahexaenoic and Arachidonic Acids as Neuroprotective Nutrients throughout the Life Cycle. Nutrients.

[43] Collins C.T., Gibson R.A., McPhee A.J., Makrides M. (2019). The Role of Long Chain Polyunsaturated Fatty Acids in Perinatal Nutrition. Semin. Perinatol..

[44] Comitini F., Peila C., Fanos V., Coscia A. (2020). The Docosahexanoic Acid: From the Maternal-Fetal Dyad to Early Life Toward Metabolomics. Front. Pediatr..

[45] Echeverría F., Valenzuela R., Catalina Hernandez-Rodas M., Valenzuela A. (2017). Docosahexaenoic Acid (DHA), a Fundamental Fatty Acid for the Brain: New Dietary Sources. Prostaglandins Leukot. Essent. Fat. Acids.

[46] Pettoello-Mantovani M., Bali D., Sevketoglu E., Pastore M., Vural M., Giardino I. (2025). The First Thousand Days: Nourishing the Developing Brain for a Lifetime of Mental Well-Being. Narrative Review. Glob. Pediatr..

[47] Li J., Pora B.L.R., Dong K., Hasjim J. (2021). Health Benefits of Docosahexaenoic Acid and Its Bioavailability: A Review. Food Sci. Nutr..

[48] Gould J.F., Roberts R.M., Makrides M. (2021). The Influence of Omega-3 Long-Chain Polyunsaturated Fatty Acid, Docosahexaenoic Acid, on Child Behavioral Functioning: A Review of Randomized Controlled Trials of Dha Supplementation in Pregnancy, the Neonatal Period and Infancy. Nutrients.

[49] Beluska-Turkan K., Korczak R., Hartell B., Moskal K., Maukonen J., Alexander D.E., Salem N., Harkness L., Ayad W., Szaro J. (2019). Nutritional Gaps and Supplementation in the First 1000 Days. Nutrients.

[50] Cusick S.E., Georgieff M.K. (2016). The Role of Nutrition in Brain Development: The Golden Opportunity of the “First 1000 Days.”. J. Pediatr..

[51] Duttaroy A.K., Basak S. (2020). Maternal Dietary Fatty Acids and Their Roles in Human Placental Development. Prostaglandins Leukot. Essent. Fat. Acids.

[52] Basak S., Mallick R., Banerjee A., Pathak S., Duttaroy A.K. (2021). Maternal Supply of Both Arachidonic and Docosahexaenoic Acids Is Required for Optimal Neurodevelopment. Nutrients.

[53] Devarshi P.P., Grant R.W., Ikonte C.J., Mitmesser S.H. (2019). Maternal Omega-3 Nutrition, Placental Transfer and Fetal Brain Development in Gestational Diabetes and Preeclampsia. Nutrients.

[54] Muñoz Y., Mercado L., Farias C., Beyer M.P., Alvear I., Echeverría F., Valenzuela R. (2024). Impact of Polyunsaturated Fatty Acids during and Pregnancy and Lactation: A Comprehensive Review. Prostaglandins Leukot. Essent. Fat. Acids.

[55] Miles E.A., Childs C.E., Calder P.C. (2021). Long-Chain Polyunsaturated Fatty Acids (LCPUFAs) and the Developing Immune System: A Narrative Review. Nutrients.

[56] Sun G.Y., Simonyi A., Fritsche K.L., Chuang D.Y., Hannink M., Gu Z., Greenlief C.M., Yao J.K., Lee J.C., Beversdorf D.Q. (2018). Docosahexaenoic Acid (DHA): An Essential Nutrient and a Nutraceutical for Brain Health and Diseases. Prostaglandins Leukot. Essent. Fat. Acids.

[57] Díaz M., Mesa-Herrera F., Marín R. (2021). Dha and Its Elaborated Modulation of Antioxidant Defenses of the Brain: Implications in Aging and Ad Neurodegeneration. Antioxidants.

[58] Lafuente M., Rodríguez González-Herrero M.E., Romeo Villadóniga S., Domingo J.C. (2021). Antioxidant Activity and Neuroprotective Role of Docosahexaenoic Acid (DHA) Supplementation in Eye Diseases That Can Lead to Blindness: A Narrative Review. Antioxidants.

[59] Hansen S., Strøm M., Maslova E., Dahl R., Hoffmann H.J., Rytter D., Bech B.H., Henriksen T.B., Granström C., Halldorsson T.I. (2017). Fish Oil Supplementation during Pregnancy and Allergic Respiratory Disease in the Adult Offspring. J. Allergy Clin. Immunol..

[60] Best K.P., Gold M., Kennedy D., Martin J., Makrides M. (2016). Omega-3 Long-Chain PUFA Intake during Pregnancy and Allergic Disease Outcomes in the Offspring: A Systematic Review and Meta-Analysis of Observational Studies and Randomized Controlled Trials. Am. J. Clin. Nutr..

[61] Richard C., Lewis E.D., Field C.J. (2016). Evidence for the Essentiality of Arachidonic and Docosahexaenoic Acid in the Postnatal Maternal and Infant Diet for the Development of the Infant’s Immune System Early in Life. Appl. Physiol. Nutr. Metab..

[62] Shrestha N., Sleep S.L., Cuffe J.S.M., Holland O.J., Perkins A.V., Yau S.Y., McAinch A.J., Hryciw D.H. (2020). Role of Omega-6 and Omega-3 Fatty Acids in Fetal Programming. Clin. Exp. Pharmacol. Physiol..

[63] Feltham B.A., Louis X.L., Eskin M.N.A., Suh M. (2020). Docosahexaenoic Acid: Outlining the Therapeutic Nutrient Potential to Combat the Prenatal Alcohol-Induced Insults on Brain Development. Adv. Nutr..

[64] Serrano M., Rico-Barrio I., Grandes P. (2023). The Effect of Omega-3 Fatty Acids on Alcohol-Induced Damage. Front. Nutr..

[65] European Food Safety Authority (EFSA) (2017). Dietary Reference Values for Nutrients Summary Report. EFSA Supporting Publications.

[66] SINU (Società Italiana Di Nutrizione Umana) (2024). LARN-Livelli Assunzione di Riferimento di Nutrienti ed Energia per La Italiana.

[67] Fondazione Confalonieri Ragonese (2018). Nutrizione in Gravidanza E Durante L’Allattamento.

[68] Likhar A., Patil M.S. (2022). Importance of Maternal Nutrition in the First 1,000 Days of Life and Its Effects on Child Development: A Narrative Review. Cureus.

[69] Massari M., Novielli C., Mandò C., Di Francesco S., Della Porta M., Cazzola R., Panteghini M., Savasi V., Maggini S., Schaefer E. (2020). Multiple Micronutrients and Docosahexaenoic Acid Supplementation during Pregnancy: A Randomized Controlled Study. Nutrients.

[70] Khor G.L. (2022). Implications of Maternal Diet on Breast Milk Docosahexaenoic Acid 22:6n-3 (DHA) and Arachidonic Acid 20:4n-6 (AA) Contents: A Narrative Review. Asia Pac. J. Clin. Nutr..

[71] Hibbeln C.J.R., Spiller P., Brenna J.T., Golding J., Holub B.J., Harris W.S., Kris-Etherton P., Lands B., Connor S.L., Myers G. (2019). Relationships between Seafood Consumption during Pregnancy and Childhood and Neurocognitive Development: Two Systematic Reviews. Prostaglandins Leukot. Essent. Fat. Acids.

[72] Nevins J.E.H., Donovan S.M., Snetselaar L., Dewey K.G., Novotny R., Stang J., Taveras E.M., Kleinman R.E., Bailey R.L., Raghavan R. (2021). Omega-3 Fatty Acid Dietary Supplements Consumed during Pregnancy and Lactation and Child Neurodevelopment: A Systematic Review. J. Nutr..

[73] Abdelrahman M.A., Osama H., Saeed H., Madney Y.M., Harb H.S., Abdelrahim M.E.A. (2023). Impact of N-3 Polyunsaturated Fatty Acid Intake in Pregnancy on Maternal Health and Birth Outcomes: Systematic Review and Meta-Analysis from Randomized Controlled Trails. Arch. Gynecol. Obstet..

[74] Bakouei F., Delavar M.A., Mashayekh-Amiri S., Esmailzadeh S., Taheri Z. (2020). Efficacy of N-3 Fatty Acids Supplementation on the Prevention of Pregnancy Induced-Hypertension or Preeclampsia: A Systematic Review and Meta-Analysis. Taiwan. J. Obstet. Gynecol..

[75] Middleton P., Gomersall J.C., Gould J.F., Shepherd E., Olsen S.F., Makrides M. (2018). Omega-3 Fatty Acid Addition during Pregnancy. Cochrane Database Syst. Rev..

[76] Savona-Ventura C., Mahmood T., Mukhopadhyay S., Louwen F. (2024). Omega-3 Fatty Acid Supply in Pregnancy for Risk Reduction of Preterm and Early Preterm Birth: A Position Statement by the European Board and College of Obstetrics and Gynaecology (EBCOG). Eur. J. Obstet. Gynecol. Reprod. Biol..

[77] Hao Y., Sun X., Wen N., Song D., Li H. (2022). Effects of N-3 Polyunsaturated Fatty Acid Supplementation on Pregnancy Outcomes: A Systematic Review and Meta-Analysis. Arch. Med. Sci..

[78] Amza M., Haj Hamoud B., Sima R.M., Dinu M.D., Gorecki G.P., Popescu M., Gică N., Poenaru M.O., Pleș L. (2024). Docosahexaenoic Acid (DHA) and Eicosapentaenoic Acid (EPA)—Should They Be Mandatory Supplements in Pregnancy?. Biomedicines.

[79] Bilgundi K., Viswanatha G.L., Purushottam K.M., John J., Kamath A.P., Kishore A., Nayak P.G., Nandakumar K. (2024). Docosahexaenoic Acid and Pregnancy: A Systematic Review and Meta-Analysis of the Association with Improved Maternal and Fetal Health. Nutr. Res..

[80] Jiang Y., Chen Y., Wei L., Zhang H., Zhang J., Zhou X., Zhu S., Du Y., Su R., Fang C. (2023). DHA Supplementation and Pregnancy Complications. J. Transl. Med..

[81] Baker E.J., Calder P.C., Kermack A.J., Brown J.E., Mustapha M., Kitson-Reynolds E., Garvey J.J. (2024). Omega-3 LC-PUFA Consumption Is Now Recommended for Women of Childbearing Age and during Pregnancy to Protect against Preterm and Early Preterm Birth: Implementing This Recommendation in a Sustainable Manner. Front. Nutr..

[82] Kar S., Wong M., Rogozinska E., Thangaratinam S. (2016). Effects of Omega-3 Fatty Acids in Prevention of Early Preterm Delivery: A Systematic Review and Meta-Analysis of Randomized Studies. Eur. J. Obstet. Gynecol. Reprod. Biol..

[83] Meher A.P., Kathaley M. (2025). Maternal, Placental and Cord Erythrocyte Levels of Long-Chain Polyunsaturated Fatty Acids in Full-Term and Preterm Pregnancy. J. Neonatal. Perinatal. Med..

[84] Saccone G., Berghella V. (2015). Omega-3 Supplementation to Prevent Recurrent Preterm Birth: A Systematic Review and Metaanalysis of Randomized Controlled Trials. Am. J. Obstet. Gynecol..

[85] Best K.P., Gibson R.A., Makrides M. (2022). ISSFAL Statement Number 7—Omega-3 Fatty Acids during Pregnancy to Reduce Preterm Birth. Prostaglandins Leukot. Essent. Fat. Acids.

[86] Parisi F., Mandò C., Novielli C., Anelli G.M., Cazzola R., Lisso F., Sarno L., Marelli E., Lubrano C., Maria Antonazzo P.G. (2025). Maternal Mid-Pregnancy Long-Chain Polyunsaturated Fatty Acid Profile Is Associated with Pregestational Body Mass Index and Neonatal Anthropometric Measures at Birth among Non-Obese Pregnancies: Results from Two Italian Multicenter Cohorts. Nutr. Metab..

[87] Ren X., Vilhjálmsdóttir B.L., Rohde J.F., Walker K.C., Runstedt S.E., Lauritzen L., Heitmann B.L., Specht I.O. (2021). Systematic Literature Review and Meta-Analysis of the Relationship Between Polyunsaturated and Trans Fatty Acids During Pregnancy and Offspring Weight Development. Front. Nutr..

[88] Dewi M., Andarwulan N., Wahyuningsih U., Kazimierczak R., Średnicka-Tober D. (2025). Maternal Long-Chain Polyunsaturated Fatty Acids Status in Pregnancy and Newborn Body Composition. Nutrients.

[89] Meher A., Randhir K., Mehendale S., Wagh G., Joshi S. (2016). Maternal Fatty Acids and Their Association with Birth Outcome: A Prospective Study. PLoS ONE.

[90] Kadam V., Dangat K., Pisal H., Randhir K., Mehendale S., Deshpande R., Lalwani S., Fall C., Joshi S. (2025). Maternal Plasma Fatty Acids at Delivery Are Associated with Newborn Weight and Anthropometric Measures in Children at 3–7 Years. Nutr. Metab. Cardiovasc. Dis..

[91] Ramakrishnan U., Gonzalez-Casanova I., Schnaas L., DiGirolamo A., Quezada A.D., Pallo B.C., Hao W., Neufeld L.M., Rivera J.A., Stein A.D. (2016). Prenatal Supplementation with DHA Improves Attention at 5 y of Age: A Randomized Controlled Trial. Am. J. Clin. Nutr..

[92] Sass L., Bjarnadóttir E., Stokholm J., Chawes B., Vinding R.K., Mora-Jensen A.R.C., Thorsen J., Noergaard S., Ebdrup B.H., Jepsen J.R.M. (2021). Fish Oil Supplementation in Pregnancy and Neurodevelopment in Childhood—A Randomized Clinical Trial. Child Dev..

[93] Tarui T., Rasool A., O’Tierney-Ginn P. (2022). How the Placenta-Brain Lipid Axis Impacts the Nutritional Origin of Child Neurodevelopmental Disorders: Focus on Attention Deficit Hyperactivity Disorder and Autism Spectrum Disorder. Exp. Neurol..

[94] Colombo J., Shaddy D.J., Mathis N., Christifano D.N., Brown A.R., Gajewski B.J., Carlson S.E., Gustafson K.M. (2025). Effects of Prenatal DHA Dose on Infant Visual Attention. Dev. Psychobiol..

[95] Mun J.G., Legette L.L., Ikonte C.J., Mitmesser S.H. (2019). Choline and DHA in Maternal and Infant Nutrition: Synergistic Implications in Brain and Eye Health. Nutrients.

[96] Khandelwal S., Swamy M.K., Patil K., Kondal D., Chaudhry M., Gupta R., Divan G., Kamate M., Ramakrishnan L., Bellad M.B. (2018). The Impact of DocosaHexaenoic Acid Supplementation during Pregnancy and Lactation on Neurodevelopment of the Offspring in India (DHANI): Trial Protocol. BMC Pediatr..

[97] Gustafson K.M., Christifano D.N., Hoyer D., Schmidt A., Carlson S.E., Colombo J., Mathis N.B., Sands S.A., Chollet-Hinton L., Brown A.R. (2022). Prenatal Docosahexaenoic Acid Effect on Maternal-Infant DHA-Equilibrium and Fetal Neurodevelopment: A Randomized Clinical Trial. Pediatr. Res..

[98] Meldrum S., Dunstan J.A., Foster J.K., Simmer K., Prescott S.L. (2015). Maternal Fish Oil Supplementation in Pregnancy: A 12 Year Follow-up of a Randomised Controlled Trial. Nutrients.

[99] Gawlik N.R., Anderson A.J., Makrides M., Kettler L., Gould J.F. (2020). The Influence of DHA on Language Development: A Review of Randomized Controlled Trials of DHA Supplementation in Pregnancy, the Neonatal Period, and Infancy. Nutrients.

[100] Guillot M., Synnes A., Pronovost E., Qureshi M., Daboval T., Caouette G., Olivier F., Bartholomew J., Mohamed I., Masse E. (2022). Maternal High-Dose DHA Supplementation and Neurodevelopment at 18–22 Months of Preterm Children. Pediatrics.

[101] Shahabi B., Hernández-Martínez C., Jardí C., Aparicio E., Arija V. (2025). Maternal Omega-6/Omega-3 Concentration Ratio During Pregnancy and Infant Neurodevelopment: The ECLIPSES Study. Nutrients.

[102] Bragg M.G., Prado E.L., Stewart C.P. (2022). Choline and Docosahexaenoic Acid during the First 1000 Days and Children’s Health and Development in Low- and Middle-Income Countries. Nutr. Rev..

[103] Azaryah H., Verdejo-Román J., Martin-Pérez C., García-Santos J.A., Martínez-Zaldívar C., Torres-Espínola F.J., Campos D., Koletzko B., Pérez-García M., Catena A. (2020). Effects of Maternal Fish Oil and/or 5-Methyl-Tetrahydrofolate Supplementation during Pregnancy on Offspring Brain Resting-State at 10 Years Old: A Follow-up Study from the Nuheal Randomized Controlled Trial. Nutrients.

[104] Rodriguez-Santana Y., Ochoa J.J., Lara-Villoslada F., Kajarabille N., Saavedra-Santana P., Hurtado J.A., Peña M., Diaz-Castro J., Sebastian-Garcia I., Machin-Martin E. (2017). Cytokine Distribution in Mothers and Breastfed Children after Omega-3 LCPUFAs Supplementation during the Last Trimester of Pregnancy and the Lactation Period: A Randomized, Controlled Trial. Prostaglandins Leukot. Essent. Fat. Acids.

[105] Valentine C.J., Dingess K.A., Kleiman J., Morrow A.L., Rogers L.K. (2019). A Randomized Trial of Maternal Docosahexaenoic Acid Supplementation to Reduce Inflammation in Extremely Preterm Infants. J. Pediatr. Gastroenterol. Nutr..

[106] Chercoles E.R. (2017). Fish Oil-Derived Fatty Acids in Pregnancy and Wheeze and Asthma in Offspring. Acta Pediatr. Esp..

[107] Henry C.O., Allsopp P.J., Yeates A.J., Spence T., Conway M.C., Mulhern M.S., Shroff E., Shamlaye C.F., Henderson J., van Wijngaarden E. (2025). Associations between Maternal Fish Intake and Polyunsaturated Fatty Acid Status with Childhood Asthma in a High Fish-Eating Population. Pediatr. Allergy Immunol..

[108] Yalagala P.C.R., Sugasini D., Chantapim S., Caal K., Sun H., Nicastro S., Sargis R.M., Gregg B., Subbaiah P.V. (2025). Efficient Enrichment of Docosahexaenoic Acid (DHA) in Mother’s Milk and in the Brain and Retina of the Offspring by Lysophosphatidylcholine (LPC)-DHA in the Maternal Diet. Nutrients.

[109] ASPEN (2024). Nutrition in the First 1000 Days: Deficiency and Toxicity Prevention for Cognitive Development.

[110] Liu B., Liu Y., Zhai C., Wu X., Wang Y., Fang X. (2025). The Multifaceted Roles of Fatty Acids and Their Dysregulation in Obese Mothers: Potential Implications for Infant Development. Nutr. Metab..

[111] Mazurier E., Rigourd V., Perez P., Buffin R., Couedelo L., Vaysse C., Belcadi W., Sitta R., Nacka F., Lamireau D. (2017). Effects of Maternal Supplementation with Omega-3 Precursors on Human Milk Composition. J. Hum. Lact..

[112] He T., Zhang J., Zhao A., Luo S., Jiang H., Tang M., Zhang Y. (2025). Docosahexaenoic Acid Supplementation in Pregnancy and Early Lactation: Impacts on Breast Milk Docosahexaenoic Acid and Maternal–Infant Gut Microbiota—A Randomized Controlled Trial. Clin. Nutr..

[113] Ueno H.M., Higurashi S., Shimomura Y., Wakui R., Matsuura H., Shiota M., Kubouchi H., Yamamura J.-I., Toba Y., Kobayashi T. (2020). Original Research Maternal and Pediatric Nutrition Association of DHA Concentration in Human Breast Milk with Maternal Diet and Use of Supplements: A Cross-Sectional Analysis of Data from the Japanese Human Milk Study Cohort.

[114] Smith S.L., Rouse C.A. (2017). Docosahexaenoic Acid and the Preterm Infant. Matern. Health Neonatol. Perinatol..

[115] Cormack B.E., Harding J.E., Miller S.P., Bloomfield F.H. (2019). The Influence of Early Nutrition on Brain Growth and Neurodevelopment in Extremely Preterm Babies: A Narrative Review. Nutrients.

[116] Silveira R.C., Corso A.L., Procianoy R.S. (2023). The Influence of Early Nutrition on Neurodevelopmental Outcomes in Preterm Infants. Nutrients.

[117] Baack M.L., Puumala S.E., Messier S.E., Pritchett D.K., Harris W.S. (2016). Daily Enteral DHA Supplementation Alleviates Deficiency in Premature Infants. Lipids.

[118] Frost B.L., Patel A.L., Robinson D.T., Berseth C.L., Cooper T., Caplan M. (2021). Randomized Controlled Trial of Early Docosahexaenoic Acid and Arachidonic Acid Enteral Supplementation in Very Low Birth Weight Infants. J. Pediatr..

[119] Marc I., Lavoie P.M., Sullivan T.R., Pronovost E., Boutin A., Beltempo M., Guillot M., Gould J.F., Simonyan D., McPhee A.J. (2025). High-Dose Docosahexaenoic Acid for Bronchopulmonary Dysplasia Severity in Very Preterm Infants: A Collaborative Individual Participant Data Meta-Analysis. Am. J. Clin. Nutr..

[120] Bernabe-García M., Calder P.C., Villegas-Silva R., Rodríguez-Cruz M., Chávez-Sánchez L., Cruz-Reynoso L., Mateos-Sánchez L., Lara-Flores G., Aguilera-Joaquín A.R., Sánchez-García L. (2021). Efficacy of Docosahexaenoic Acid for the Prevention of Necrotizing Enterocolitis in Preterm Infants: A Randomized Clinical Trial. Nutrients.

[121] Abou El Fadl D.K., Ahmed M.A., Aly Y.A., Darweesh E.A.G., Sabri N.A. (2021). Impact of Docosahexaenoic Acid Supplementation on Proinflammatory Cytokines Release and the Development of Necrotizing Enterocolitis in Preterm Neonates: A Randomized Controlled Study: Impact of Docosahexaenoic Acid Supplementation on Proinflammatory Cytokines. Saudi Pharm. J..

[122] Alshaikh B.N., Reyes Loredo A., Yusuf K., Maarouf A., Fenton T.R., Momin S. (2023). Enteral Long-Chain Polyunsaturated Fatty Acids and Necrotizing Enterocolitis: A Systematic Review and Meta-Analysis. Am. J. Clin. Nutr..

[123] Malikiwi A.I., Lee Y.M., Davies-Tuck M., Wong F.Y. (2019). Postnatal Nutritional Deficit Is an Independent Predictor of Bronchopulmonary Dysplasia among Extremely Premature Infants Born at or Less than 28 weeks Gestation. Early Hum. Dev..

[124] Wendel K., Aas M.F., Gunnarsdottir G., Rossholt M.E., Bratlie M., Nordvik T., Landsend E.C.S., Fugelseth D., Domellöf M., Pripp A.H. (2023). Effect of Arachidonic and Docosahexaenoic Acid Supplementation on Respiratory Outcomes and Neonatal Morbidities in Preterm Infants. Clin. Nutr..

[125] Marc I., Piedboeuf B., Lacaze-Masmonteil T., Fraser W., Mâsse B., Mohamed I., Qureshi M., Afifi J., Lemyre B., Caouette G. (2020). Effect of Maternal Docosahexaenoic Acid Supplementation on Bronchopulmonary Dysplasia–Free Survival in Breastfed Preterm Infants. JAMA.

[126] Collins C.T., Makrides M., McPhee A.J., Sullivan T.R., Davis P.G., Thio M., Simmer K., Rajadurai V.S., Travadi J., Berry M.J. (2017). Docosahexaenoic Acid and Bronchopulmonary Dysplasia in Preterm Infants. N. Engl. J. Med..

[127] Dang D., Gao Z., Zhang C., Mu X., Lv X., Wu H. (2025). Effect of Enteral Supplementation of DHA with or without ARA in Preterm Infants: A Meta-Analysis. Arch. Dis. Child.-Fetal Neonatal Ed..

[128] Pivodic A., Johansson H., Smith L.E.H., Löfqvist C., Albertsson-Wikland K., Nilsson S., Hellström A. (2022). Evaluation of the Retinopathy of Prematurity Activity Scale (ROP-ActS) in a Randomised Controlled Trial Aiming for Prevention of Severe ROP: A Substudy of the Mega Donna Mega Trial. BMJ Open Ophthalmol..

[129] Bernabe-García M., Villegas-Silva R., Villavicencio-Torres A., Calder P.C., Rodríguez-Cruz M., Maldonado-Hernández J., Macías-Loaiza D., López-Alarcón M., Inda-Icaza P., Cruz-Reynoso L. (2019). Enteral Docosahexaenoic Acid and Retinopathy of Prematurity: A Randomized Clinical Trial. J. Parenter. Enter. Nutr..

[130] Hellström A., Pivodic A., Gränse L., Lundgren P., Sjöbom U., Nilsson A.K., Söderling H., Hård A.L., Smith L.E.H., Löfqvist C.A. (2021). Association of Docosahexaenoic Acid and Arachidonic Acid Serum Levels with Retinopathy of Prematurity in Preterm Infants. JAMA Netw. Open.

[131] Moltu S.J., Nordvik T., Rossholt M.E., Wendel K., Chawla M., Server A., Gunnarsdottir G., Pripp A.H., Domellöf M., Bratlie M. (2024). Arachidonic and Docosahexaenoic Acid Supplementation and Brain Maturation in Preterm Infants; a Double Blind RCT. Clin. Nutr..

[132] Hewawasam E., Collins C.T., Muhlhausler B.S., Yelland L.N., Smithers L.G., Colombo J., Makrides M., McPhee A.J., Gould J.F. (2021). DHA Supplementation in Infants Born Preterm and the Effect on Attention at 18 Months’ Corrected Age: Follow-up of a Subset of the N3RO Randomised Controlled Trial. Br. J. Nutr..

[133] Paquet S.P., Pronovost E., Simonyan D., Caouette G., Matte-Gagné C., Olivier F., Bartholomew J., Morin A., Mohamed I., Marc I. (2024). Maternal High-Dose Docosahexaenoic Acid Supplementation and Neurodevelopment at 5 Years of Preterm Children. Clin. Nutr. ESPEN.

[134] Andrew M.J., Parr J.R., Montague-Johnson C., Laler K., Holmes J., Baker B., Sullivan P.B. (2018). Neurodevelopmental Outcome of Nutritional Intervention in Newborn Infants at Risk of Neurodevelopmental Impairment: The Dolphin Neonatal Double-Blind Randomized Controlled Trial. Dev. Med. Child Neurol..

[135] Gunnarsdottir G., Rossholt M.E., Nordvik T., Wendel K., Aas M.F., Skarbø A.B., Aulie V.S., Stiris T., Ramm-Pettersen A., Pfeiffer H.C. (2025). High Dose Arachidonic and Docosahexaenoic Acid in Very Preterm Infants and Neurodevelopment at 2 Years—A Double-Blind Randomized Controlled Trial. Clin. Nutr..

[136] Liu Y., Zhang G., Chen T., Kong H., Huang S. (2025). Early Long-Chain Polyunsaturated Fatty Acids Supplementation on Long- and Short-Term Neurodevelopmental Outcomes in Preterm or Low Birth Weight Infants: A Meta-Analysis. Matern. Child Nutr..

[137] Gould J.F., Makrides M., Gibson R.A., Sullivan T.R., McPhee A.J., Anderson P.J., Best K.P., Sharp M., Cheong J.L.Y., Opie G.F. (2022). Neonatal Docosahexaenoic Acid in Preterm Infants and Intelligence at 5 Years. N. Engl. J. Med..

[138] Shepherd E., Ikeda N., Sullivan T.R., Marc I., Guillot M., McPhee A.J., Gibson R.A., Makrides M., Gould J.F. (2025). Enteral High-Dose Docosahexaenoic Acid and Neurodevelopment in Extremely Preterm Infants: A Systematic Review and Meta-Analysis. Curr. Dev. Nutr..

[139] Suganuma H., Ikeda N., Ohkawa N., Kaga N., Taka H., Miura Y., Shoji H. (2025). Association between Polyunsaturated Fatty Acid Intake and Plasma Oxylipin Profiles in Preterm Infants: A Retrospective Cohort Study. J. Parenter. Enter. Nutr..

[140] Mozurkewich E.L., Greenwood M., Clinton C., Berman D., Romero V., Djuric Z., Qualls C., Gronert K. (2016). Pathway Markers for Pro-Resolving Lipid Mediators in Maternal and Umbilical Cord Blood: A Secondary Analysis of the Mothers, Omega-3, and Mental Health Study. Front. Pharmacol..

[141] Carlson S.E., Colombo J. (2021). DHA and Cognitive Development. J. Nutr..

[142] Tabilo C., Squella V., Illesca P., Muñoz Y., Farías C., Valenzuela R. (2025). Impact of N-3 Polyunsaturate Fatty Acids Supplementation on Visual Health throughout the Life Cycle: A Systematic Review. Prostaglandins Leukot. Essent. Fat. Acids.

[143] Shulkin M., Pimpin L., Bellinger D., Kranz S., Fawzi W., Duggan C., Mozaffarian D. (2018). N-3 Fatty Acid Supplementation in Mothers, Preterm Infants, and Term Infants and Childhood Psychomotor and Visual Development: A Systematic Review and Meta-Analysis. J. Nutr..

[144] Andrew M.J., Parr J.R., Montague-Johnson C., Laler K., Qi C., Baker B., Sullivan P.B. (2018). Nutritional Intervention and Neurodevelopmental Outcome in Infants with Suspected Cerebral Palsy: The Dolphin Infant Double-Blind Randomized Controlled Trial. Dev. Med. Child Neurol..

[145] Hu R., Xu J., Hua Y., Li Y., Li J. (2024). Could Early Life DHA Supplementation Benefit Neurodevelopment? A Systematic Review and Meta-Analysis. Front. Neurol..

[146] Lehner A., Staub K., Aldakak L., Eppenberger P., Rühli F., Martin R.D., Bender N. (2021). Impact of Omega-3 Fatty Acid DHA and EPA Supplementation in Pregnant or Breast-Feeding Women on Cognitive Performance of Children: Systematic Review and Meta-Analysis. Nutr. Rev..

[147] Foiles A.M., Kerling E.H., Wick J.A., Scalabrin D.M.F., Colombo J., Carlson S.E. (2016). Formula with Long-Chain Polyunsaturated Fatty Acids Reduces Incidence of Allergy in Early Childhood. Pediatr. Allergy Immunol..

[148] Smilowitz J.T., Allen L.H., Dallas D.C., McManaman J., Raiten D.J., Rozga M., Sela D.A., Seppo A., Williams J.E., Young B.E. (2023). Ecologies, Synergies, and Biological Systems Shaping Human Milk Composition—A Report from “Breastmilk Ecology: Genesis of Infant Nutrition (BEGIN)” Working Group 2. Am. J. Clin. Nutr..

[149] Okai-Mensah P., Brkić D., Hauser J. (2025). The Importance of Lipids for Neurodevelopment in Low and Middle Income Countries. Front. Nutr..

[150] Andrew M.J., Embleton N., Hardy P., Johnson S., Juszczak E., Ledbury H., Pearson C., Rivero-Arias O., Francisco A.A., Berrington J. (2025). Trial Protocol: DOLFIN Trial: Developmental Outcomes of Long-Term Feed Supplementation in Neonates—A UK Multicentre, Blinded, Stratified, Randomised Controlled Trial. Trials.

[151] Janson E., Koolschijn P.C.M.P., Schipper L., Boerma T.D., Wijnen F.N.K., de Boode W.P., van den Akker C.H.P., Licht-van der Stap R.G., Nuytemans D.H.G.M., Onland W. (2024). Dolphin CONTINUE: A Multi-Center Randomized Controlled Trial to Assess the Effect of a Nutritional Intervention on Brain Development and Long-Term Outcome in Infants Born before 30 Weeks of Gestation. BMC Pediatr..

