# Supplementary material for: DHA: Nutritional Programming During the First 1000 Days of Life
Source: Nutrients. 2026 Apr 9;18(8):1178. doi: 10.3390/nu18081178 (PMC13118849; doi:10.3390/nu18081178)
Supplement: Supplementary file 1 [file nutrients-18-01178-s001.zip › nutrients-4181288-supplementary.pdf]

Table S1. *DHA during pregnancy and lactation*

First author, year	Ref.	Study type	Population	DHA dose / intervention	Co-nutrients	Key outcomes	Main findings	LOE
**Massari, 2020**	[69]	RCT	Pregnant women	200 mg/day DHA from 13–15 weeks to delivery	Multiple micronutrients	Maternal DHA status	Improved maternal DHA status; limited by lack of placebo and blinding	**Moderate**
**Khor, 2022**	[70]	Narrative review	Pregnant/lactating women	Dietary DHA exposure	None	Breast milk DHA/ARA, infant growth/development implications	Breast milk DHA reflects maternal diet; low fish intake may worsen n-6/n-3 balance	**Low**
**Hibbeln, 2019**	[71]	Systematic review	Pregnant women / offspring	Dietary fish/seafood intake	None	Neurocognitive development	Maternal seafood intake associated with better neurocognitive outcomes	**Moderate**
**Nevins, 2021**	[72]	Systematic review	Pregnancy/lactation	Variable	Variable	Language, motor, socio-emotional, neuropsychiatric outcomes	Evidence insufficient/inconsistent	**Moderate**
**Abdelrahman, 2023**	[73]	Systematic review/meta-analysis	Pregnant women	Variable	EPA ± DHA	Maternal health, birth outcomes	Supports beneficial effects for some pregnancy outcomes	**Moderate**
**Bakouei, 2020**	[74]	Systematic review/meta-analysis	Pregnant women	Variable	EPA ± DHA	PIH/preeclampsia	Suggests preventive benefit in some datasets	**Moderate**
**Middleton, 2018**	[75]	Cochrane review	Pregnant women	Variable	EPA ± DHA	Pregnancy outcomes, preterm birth	Mixed evidence; some reduction in preterm birth	**High–Moderate**
**Savona-Ventura, 2024**	[76]	Position statement	Pregnant women	n-3 PUFA	None	Preterm birth policy implications	Supports omega-3 intake in pregnancy	**Low**
**Hao, 2022**	[77]	Systematic review/meta-analysis	Pregnant women	Variable	EPA ± DHA	Pregnancy outcomes	Mixed but overall favorable trends for some outcomes	**Moderate**
**Amza, 2024**	[78]	Narrative review	Pregnant women	Variable	EPA	Maternal/fetal outcomes	Supports supplementation	**Low**
**Bilgundi, 2024**	[79]	Systematic review/meta-analysis	Pregnant women	Variable	EPA ± DHA	Maternal and fetal health	Suggests overall benefit, depending on outcome	**Moderate**
**Jiang, 2023**	[80]	Review	Pregnant women	Variable	None/variable	Pregnancy complications	Supports biological plausibility and possible benefit	**Low**

**Baker, 2024**	[81]	Narrative review	Women of childbearing age, pregnant women	Dietary intake emphasis	EPA ± DHA	Preterm birth prevention	Supports implementation of recommendations	**Low**
**Kar, 2016**	[82]	Systematic review/meta-analysis	Pregnant women	Variable	EPA ± DHA	Early preterm birth	Suggests benefit for early preterm birth	**Moderate**
**Meher, 2025**	[83]	Observational study	Pregnant women	Status study	None	Maternal/placental/cord fatty acids	Links maternal/placental PUFA status with birth outcomes	**Low**
**Saccone, 2015**	[84]	Systematic review/meta-analysis	Pregnant women with prior preterm birth	Variable	EPA ± DHA	Recurrent preterm birth	No consistent reduction in preterm birth	**Moderate**
**Best, 2022**	[85]	Position statement	Pregnant women	200 mg/day DHA	None	Maternal/placental/fetal DHA status, preterm birth	200 mg/day may be insufficient in normal-weight women, but may increase RBC DHA in high-BMI women	**Low–Moderate**
**Parisi, 2025**	[86]	Secondary analysis	Non-obese pregnancies	Status study	None	Maternal BMI, neonatal anthropometry	Maternal LCPUFA profile associated with anthropometry	**Low**
**Ren, 2021**	[87]	Systematic review/meta-analysis	Pregnancy cohorts/trials	~650 mg/day	EPA ± DHA	Birth weight, childhood weight	Positive relationship, stronger at higher doses	**Moderate**
**Dewi, 2025**	[88]	Observational study	Pregnant women at 3rd trimester	Dietary intake/status	None	Neonatal adiposity/body composition	Higher DHA intake linked to lower infant fat mass	**Low**
**Meher, 2016**	[89]	Prospective observational study	Pregnant women	DHA status study	None	Birth weight	Higher maternal DHA associated with higher birth weight; LBW linked to higher n-6/ARA	**Low**
**Kadam, 2025**	[90]	Observational study	Pregnant women	LCPUFAs status study	None	Birth size, adiposity at 3–7 y	Higher maternal n-6/n-3 ratio associated with greater offspring adiposity	**Low**
**Ramakrishnan, 2016**	[91]	Placebo-controlled RCT	Pregnant women	400 mg/day DHA from 18–22 weeks to delivery	None	Attention, cognition, behavior at 5 y	Improved sustained attention; not broad cognition	**Moderate**
**Sass, 2021**	[92]	Double-blind RCT	736 mother–child pairs	n-3 LCPUFA supplementation (dose as trial protocol)	EPA + DHA	Motor, cognition, language, behavioral outcomes	Benefits in selected domains; sex-specific effects	**Moderate**

**Tarui, 2022**	[93]	Narrative review	Maternal obesity / offspring	Status/supplementation review	None	ADHD, ASD, placental transfer	Low maternal DHA and obesity linked to higher neurodevelopmental risk	**Low**
**Gould, 2021**	[48]	Systematic review	Pregnancy/neonatal/infancy RCTs	Variable	Variable	Behavioral outcomes	No consistent benefit; some adverse behavioral signals in large trials	**Moderate**
**Colombo, 2025**	[94]	RCT	Pregnant women / infants	800 vs 200 mg/day DHA	None	Visual attention at 4 and 6 months	Higher dose may benefit early visual attention; attrition limitation	**Moderate**
**Mun, 2019**	[95]	Narrative review	Pregnancy/lactation	DHA + choline	Choline	Brain/eye health	Suggests synergistic roles	**Low**
**Khandelwal, 2018**	[96]	RCT	Pregnant women (≤20 weeks) and infants (follow-up)	400 mg/day DHA (pregnancy to 6 months postpartum)	None	Neurodevelopment (DASII at 6–12 months)	Improved neurodevelopment at 12 months with sustained pre- and postnatal DHA supplementation	Moderate
**Gustafson, 2022**	[97]	Randomized double-blind RCT	Pregnant women	800 vs 200 mg/day DHA	None	Maternal–infant DHA equilibrium, fetal neurodevelopment	Dose-dependent biochemical improvement, no fetal neurodevelopmental gain	**Moderate**
**Meldrum, 2015**	[98]	Follow-up of RCT	Mother–child cohort	Fish oil in pregnancy	EPA + DHA	Cognitive/behavioral outcomes at 12 y	No significant long-term effects	**Moderate**
**Gawlik, 2020**	[99]	Systematic review	Pregnancy/neonatal/infancy studies	Variable	Variable	Language outcomes	Limited benefit; few positive trials, mostly from same study	**Moderate**
**Guillot, 2022**	[100]	Follow-up of RCT	Lactating mothers of infants <29 weeks	High-dose maternal DHA-rich algal oil until 36 weeks PMA	None	Bayley-III at 18–22 months CA	No overall benefit; possible language benefit in <27 weeks subgroup	**Moderate**
**Shahabi, 2025**	[101]	Follow-up study of RCT	Mother–infant pairs	Variable	None	BSID-III motor development	High 3rd-trimester n-6/n-3 ratio associated with poorer motor outcomes	**Low**
**Herrera, 2023**	[25]	Narrative review	Pregnancy/neonates	Recommends ≥300 mg/day pregnancy, 200 mg/day lactation	Includes ARA context	Brain/retinal development, growth	Supports DHA+ARA relevance; warns about	**Low**

							deficiency and excess	
**Bragg, 2022**	[102]	Narrative review	LMIC populations	DHA + choline	Choline	Child health/development	Supports multimodal nutritional strategies	**Low**
**Azaryah, 2020**	[103]	Follow-up of RCT	Offspring at 9.5–10 years	Maternal fish oil during pregnancy	5-MTHF	Resting-state fMRI	Fish oil associated with altered brain connectivity; clinical meaning uncertain	**Moderate**
**Rodríguez-Santana, 2017**	[104]	Double-blind RCT	46 pregnant women	320 mg/day DHA + 72 mg/day EPA from 28 weeks to 4 months postpartum	EPA	Cytokines, inflammatory profile	More anti-inflammatory cytokine pattern in mothers/infants	**Moderate**
**Valentine, 2019**	[105]	RCT	Mothers of extremely preterm infants	1000 mg/day DHA	None	Breast milk DHA, inflammatory markers	Increased milk DHA and reduced inflammatory markers	**Moderate**
**Chercoles, 2017**	[106]	Narrative review	Pregnancy/offspring	Fish oil	EPA + DHA	Wheeze/asthma	Supports reduced wheeze/asthma, especially low baseline n-3 status	**Low**
**Henry, 2025**	[107]	Observational study	High fish-eating population	Dietary intake / PUFA status	None	Childhood asthma at 7 y	Higher cord DHA associated with higher asthma prevalence; no association with maternal fish intake	**Low**
**Yalagala, 2025**	[108]	Experimental study	Maternal diet / offspring model	LPC-DHA vs TAG-DHA	None	Milk DHA, brain/retina DHA	LPC-DHA more effective in enriching milk DHA	**Low**
**Liu, 2025**	[110]	Narrative review	Obese mothers and infants	Variable	Variable	Fatty acid profile, infant development	Altered FA metabolism potentially impacts infant development	**Low**
**Mazurier, 2017**	[111]	RCT	Lactating women	ALA-enriched foods	ALA sources	Human milk fatty acids	Lower LA/ALA ratio but no increase in milk DHA	**Moderate (for milk composition outcome)**
**He, 2025**	[112]	Double-blind placebo-RCT	Pregnant/lactating women	100 mg/day DHA from 3rd trimester	None	Colostrum DHA, lactation DHA, microbiota	Increased colostrum DHA; insufficient to sustain later lactation DHA	**Moderate**
**Ueno, cohort study**	[113]	Cross-sectional study	Japanese women	Diet/supplement exposure	None	Human milk DHA	Milk DHA influenced by	**Low**

diet/supplements

Table S2. *DHA in preterm population*

First author, year	Ref.	Study type	Population	DHA dose / intervention	Co-nutrients	Key outcomes	Main findings	LOE
**Smith, 2017**	[114]	Narrative review	Preterm infants	Variable	Variable	Overview of deficiency and supplementation	Supports biological rationale for supplementation	**Low**
**Cormack, 2019**	[115]	Narrative review	Extremely preterm infants	Variable	Variable	Brain growth, neurodevelopment	Early nutrition, including LCPUFAs, is critical for brain development	**Low**
**Silveira, 2023**	[116]	Narrative review	Preterm infants	Variable	Variable	Neurodevelopment	Early-life nutrition influences neurodevelopment outcomes	**Low**
**Baack, 2016**	[117]	Double-blind RCT	Preterm infants born 24–34 weeks	50 mg/day enteral DHA	Standard nutrition	Biochemical DHA status, feasibility	Improved DHA status; levels still lower than term at discharge	**Moderate**
**Frost, 2021**	[118]	RCT	VLBW infants	DHA+ARA 120 mg vs 360 mg total LCPUFA	ARA	blood DHA concentration	Higher dose improved DHA and ARA status; well tolerated	**Moderate**
**Marc, 2025**	[119]	Meta-analysis	Infants <29 weeks	High-dose DHA (40 mg/kg/day) vs control group (no/low dose DHA)	None	Severe BPD	No increased risk of severe BPD with high dose DHA	**Moderate–High**
**Bernabe-García, 2021**	[120]	Randomized clinical trial	Preterm infants	75 mg/kg/day DHA from first enteral feed	None	NEC	Reduced NEC incidence	**Moderate**
**Abou El Fadl, 2021**	[121]	Prospective randomized controlled study	≤32 weeks, ≤1500 g neonates	DHA supplementation (dose per study)	None	IL-1β, NEC	Possible reduction in NEC and inflammatory cytokines	**Low–Moderate**
**Alshaikh, 2023**	[122]	Systematic review/meta-analysis	Preterm infants	DHA alone vs balanced LCPUFA regimens	ARA considered	NEC	DHA alone may increase NEC risk if not balanced with ARA	**Moderate**
**Malikiwi, 2019**	[123]	Observational study	Extremely preterm infants	Nutritional intake study	None	BPD/chronic lung disease	Early undernutrition	**Low**

							n predicts BPD	
**Wendel, 2023**	[124]	Secondary analysis of RCT	Preterm infants	DHA + ARA	ARA	Respiratory outcomes, neonatal morbidities	Safe; possible respiratory benefit	**Moderate**
**Marc, 2020**	[125]	Placebo-controlled RCT	Mothers of infants <29 weeks	1.2 g/day maternal DHA to 36 weeks' PMA	None	BPD-free survival	No significant improvement	**Moderate**
**Collins (N3RO), 2017**	[126]	Multicenter RCT	1273 infants <29 weeks	60 mg/kg/day DHA emulsion	None	BPD	No reduction in BPD incidence	**High**
**Dang, 2025**	[127]	Meta-analysis	Preterm infants	Enteral DHA ± ARA	ARA optional	Preterm complications incl. BPD	No protection; possible increased BPD risk	**Moderate**
**Pivodic, 2022**	[128]	RCT	Preterm infants	DHA vs no supplementation	Variable	Severe ROP	Suggests protective effect	**Low–Moderate**
**Bernabe-García, 2019**	[129]	Randomized clinical trial	Preterm infants	DHA	None	Stage 3 ROP, severe ROP	Reduced severe ROP risk	**Moderate**
**Hellström, 2021**	[130]	Observational / associative study	Very preterm infants	DHA and ARA	ARA	ROP	DHA+ARA balance associated with ROP risk	**Low**
**Moltu, 2024**	[131]	Double-blind RCT	Infants <29 weeks	DHA 50 mg/kg/day + ARA 100 mg/kg/day or MCT from the 2nd day of life to 36 weeks' PMA	ARA/MCT	DTI/TBSS white matter maturation	Improved white matter maturation	**Moderate**
**Heath, 2022**	[31]	Narrative review	Premature infants	Formula with 0.3% DHA + 0.6% ARA or LCPUFA-free formula	ARA	Neurodevelopment	Better outcomes with DHA+ARA formula	**Low**
**Hewawasam, 2021**	[132]	Follow-up of RCT	Subset of N3RO cohort	60 mg/kg/day DHA emulsion	None	Attention/distractibility at 18 months	No differences	**Moderate**
**Paquet, 2024**	[133]	Follow-up of RCT	Very preterm children	High-dose neonatal-period DHA exposure	None	Behavior, executive function	No measurable benefit at 5 years	**Moderate**
**Andrew, 2018**	[134]	Dolphin neonatal trial: double blind placebo-CT	Neonates at risk of neurodevelopmental impairment	DHA, EPA, ARA, choline, UMP, CMP, zinc, iodine, B12	Multiple nutrients, ARA	Bayley-III cognitive composite	No improved neurodevelopment in powered formula group	**Low–Moderate**
**Gunnarsdottir, 2025**	[135]	ImNuT Double-blind follow-up	Infants <29 weeks	ARA 100 mg/kg/day + DHA 50 mg/kg/day or MCT from the 2nd day of life until 36 weeks' PMA	ARA	BSID-III, PDMS-2 at 2 years CA	No significant differences	**Moderate**

**Liu, 2025**	[136]	Meta-analysis	Preterm/LBW infants	Variable LCPUFA before term	Variable	IQ, NDI, motor/language/cognitive scores	No clear IQ benefit; possible reduction in intellectual disability	**Moderate**
**Gould, 2022**	[137]	Follow-up of RCT	Children from N3RO centers	60 mg/kg/day DHA emulsion or control emulsion	None	FSIQ at 5 years CA	Modestly higher FSIQ in DHA group	**Moderate**
**Shepherd, 2025**	[138]	Systematic review/meta-analysis	Infants <29 weeks	≥40 mg/kg/day DHA or ≥0.60% DHA formula/milk	Variable	Global cognition, respiratory outcomes	No global cognition difference overall; higher cognitive scores with direct emulsion	**Moderate**
**Suganuma, 2025**	[139]	Retrospective cohort	Preterm infants	DHA and ARA	ARA	Oxylipins, lipid mediators	PUFA intake associated with oxylipin regulation	**Low**
**Mozurkewich, 2016**	[140]	Secondary analysis	Maternal/cord samples	Status markers	None	Pro-resolving mediators	Supports immune-modulatory pathways	**Low**

Table S3. *DHA during infancy and early childhood (0-2 years)*

**First author, year**	**Ref.**	**Study type**	**Population**	**DHA dose / intervention**	**Co-nutrients**	**Key outcomes**	**Main findings**	**LOE**
**Carlson, 2021**	[141]	Narrative review	Pediatric population	Variable	Variable	Cognitive development	DHA is essential for brain development, but clinical evidence remains inconsisten	**Low**
**Tabilo, 2025**	[142]	Systematic review	Infants/children	Variable	Variable	Visual outcomes	More favorable visual effects, especially in preschool and school-aged children with ADHD	**Moderate**
**Shulkin, 2018**	[143]	Systematic review/meta-analysis	Mothers, preterm and term infants	Variable	Variable	Visual acuity, MDI, PDI, IQ	Benefits most consistent for visual acuity; modest MDI/PDI gains; no clear IQ benefit	**Moderate**

**Andrew, 2018**	[144]	Double-blind RCT	Children 1–18 months with suspected cerebral palsy	DHA + choline + UMP for 2 years	Choline, UMP	Cognitive/language outcomes	No significant differences; underpowered and high withdrawal	**Low–Moderate**
**Gawlik, 2020**	[99]	Systematic review	Pregnancy/neonatal/infancy studies	Variable	Variable	Language development	Only 4 positive trials; evidence limited	**Low-Moderate**
**Hu, 2024**	[145]	Systematic review/meta-analysis	Infants, pregnancy/lactation-linked studies	Variable	Variable	MDI, PDI, neurodevelopment	No MDI difference; modest PDI benefit	**Moderate**
**Lehner, 2021**	[146]	Systematic review/meta-analysis	Children exposed prenatally/postnatally	Variable	EPA	Cognitive outcomes, birth weight	No significant association with cognitive parameters or birth weight	**Moderate**
**Foiles, 2016**	[147]	Follow-up study from DIAMOND trial	Healthy full-term infants	DHA+ARA-supplemented formula	ARA	Allergy, wheezing, asthma	Reduced allergy and respiratory illness in first year	**Moderate**
